# Risk of exacerbations, hospitalisation, and mortality in adults with physician-diagnosed chronic obstructive pulmonary disease with normal spirometry and adults with preserved ratio impaired spirometry in Sweden: retrospective analysis of data from a nationwide cohort study

**DOI:** 10.1016/j.lanepe.2025.101322

**Published:** 2025-05-14

**Authors:** Oskar Wallström, Caroline Stridsman, Helena Backman, Sigrid Vikjord, Anne Lindberg, Fredrik Nyberg, Lowie E.G.W. Vanfleteren

**Affiliations:** a Dept of Internal Medicine and Clinical Nutrition, Institute of Medicine, Sahlgrenska Academy, University of Gothenburg, Gothenburg, Sweden; b Dept of Public Health and Clinical Medicine, Umeå University, Umeå, Sweden; c Department of Public Health and Nursing, Faculty of Medicine and Health Sciences, HUNT Research Centre, NTNU, Levanger, Norway; d School of Public Health and Community Medicine, Institute of Medicine, Sahlgrenska Academy, University of Gothenburg, Gothenburg, Sweden; e Dept of Respiratory Medicine and Allergology, COPD Center, Sahlgrenska University Hospital, Gothenburg, Sweden

**Keywords:** COPD, Physician diagnosed COPD with normal spirometry, PRISm, Exacerbation, Register

## Abstract

**Background:**

Physician diagnosed COPD with normal spirometry (dnsCOPD) (sometimes labeled pre-COPD) and Preserved Ratio Impaired Spirometry (PRISm) has been studied in population-based cohorts, but not in physician diagnosed COPD (dCOPD) patients from routine clinical practice. The Swedish National Airway Register (SNAR) is a large nationwide register including data from dCOPD patients from over 1000 clinics across all regions of Sweden and is representative of the COPD care in Sweden. We aimed to identify and characterize patients with dnsCOPD, PRISm and spirometrically confirmed COPD (sCOPD) from dCOPD patients in SNAR, stratify them further according to symptoms and exacerbations risk using the Global Initiative for Chronic Obstructive Lung Disease (GOLD) A/B/E classification, and assess differences in risk for exacerbations, cause-specific hospitalisations and mortality.

**Methods:**

We enrolled patients aged ≥30 years with dCOPD in the SNAR from 1 January 2014 to 30 June 2022 with complete spirometry i.e., postbronchodilator values for both forced expiratory volume in 1 s (FEV_1_) and forced vital capacity (FVC) (index date). Patients with concomitant asthma were excluded. Patients were stratified into dnsCOPD (FEV_1_/FVC ≥0.7 and FEV_1_ ≥80% predicted), PRISm (FEV_1_/FVC ≥0.7 and FEV_1_ <80% predicted) and sCOPD (FEV_1_/FVC <0.7). Further substratification was based on GOLD A/B/E (A: COPD assessment test (CAT) score <10 points and <2 moderate, 0 severe exacerbations within 1 year before the index date, B: CAT-score ≥10 points and <2 moderate, 0 severe exacerbations, E: ≥2 moderate or ≥1 severe exacerbation(s)). Patients were followed until 31 November 2022. Competing risk regression was used to calculate subdistribution hazard ratios (SHR)s with 95% confidence intervals (CIs) for exacerbation, hospitalisation and mortality.

**Findings:**

Of 45,653 patients with dCOPD, 5.4% had dnsCOPD, 11.4% had PRISm and 83.3% had sCOPD. Smoking history was similar between groups (ever smoker: dnsCOPD: 79% PRISm: 82% sCOPD: 86%) and inhalation therapy was common in all groups (any inhaler: 75%, 80% and 80%, triple combination: 22%, 28% and 35%). Patients with PRISm had a high prevalence of obesity (dnsCOPD: 30%, PRISm: 43%, COPD: 22%), cardiovascular disease (dnsCOPD: 39%, PRISm: 48%, COPD: 41%) and diabetes (dnsCOPD: 10%, PRISm: 17%, COPD: 9%). Baseline GOLD group B or E were highly prevalent in dnsCOPD (B: 54%, E: 11%), PRISm (B: 59%, E: 14%), as well as in COPD (B: 54%, E: 17%). DnsCOPD and PRISm patients had lower risk of exacerbations (SHR 0.69, 95%CI 0.64–0.74 and 0.85, 95%CI 0.81–0.89), respiratory hospitalisation (0.40, 95%CI 0.34–0.46 and 0.68, 95%CI 0.62–0.73), and respiratory mortality (0.22, 95%CI 0.13–0.37 and 0.60, 95%CI 0.48–0.75) compared to sCOPD. Cardiovascular mortality was lower in dnsCOPD (0.41, 95%CI 0.19–0.86), but similar in PRISm (0.73, 95%CI 0.49–1.08) compared to sCOPD. The A/B/E classification was predictive for all outcomes in dnsCOPD and PRISm. DnsCOPD and PRISm group E patients had higher risks for all outcomes than sCOPD group A or B.

**Interpretation:**

DnsCOPD and PRISm are prevalent in a real-life cohort of patients with a physician diagnosis of COPD. These patients are symptomatic, might suffer from exacerbations and are commonly treated with inhaled therapy, equally to sCOPD. Patients with PRISm had a high prevalence of obesity, diabetes and cardiovascular disease. DnsCOPD and PRISm had generally lower overall risks of exacerbation or respiratory events, although PRISm patients showed similar cardiovascular risk to sCOPD. The A/B/E classification predicted future events, even in dnsCOPD and PRISm patients.

**Funding:**

This study is performed with support from The 10.13039/501100003793Swedish Heart-Lung Foundation (20200150) and the Swedish government and country council ALF grant (ALFGBG-824371).

**Research in context**

**Evidence before this study**

The term PRISm (Preserved Ratio Impaired Spirometry) has been introduced in the Global Initiative for Obstructive Lung Disease (GOLD) document to define a group of COPD patients with a normal FEV1/FVC ratio (≥0.7 post-bronchodilation) but abnormal FEV1 (<80% of predicted value). Pre-COPD similarly describes patients with a COPD diagnosis but normal spirometry, although the GOLD document mentions structural changes as well. Patients with pre-COPD or PRISm are at risk of developing substantial persistent airflow obstruction over time, although not all individuals progress, and transitions between normal and obstructed spirometry can occur over time.

The prevalence of pre-COPD and PRISm in population-based studies ranges from 7% to 20%, is higher with current and former smoking, and with both low and high body mass index, and the conditions are associated with increased mortality. Pre-COPD and PRISm are highlighted in the GOLD 2024 report as a group of patients of special interest, since they have been excluded from many studies and represent an important knowledge gap. Pre-COPD and PRISm have primarily been investigated in cohorts of (ex-) smokers like COPDGene or population-based cohorts such as The Copenhagen City Heart Study or the Rotterdam study. Previous research has established a link between PRISm and a range of disease outcomes, including diabetes, metabolic syndrome, hypertension, stroke and cardiovascular disease. The Novelty study showed that a large portion of patients exhibit unobstructed post-bronchodilator spirometry in a clinical cohort of patients with physician-diagnosed COPD.

**Added value of this study**

Our findings show that physician diagnosed COPD with normal spirometry (dnsCOPD) and PRISm are prevalent among patients with a physician diagnosis of COPD. These patients frequently experience a high symptom burden, exacerbations and receive similar treatment with inhaled therapy, as those with sCOPD. Notably, PRISm patients had a high prevalence of obesity, diabetes and cardiovascular disease. While both dnsCOPD and PRISm had generally lower overall risks of exacerbation or respiratory events, PRISm patients showed similar cardiovascular risk to sCOPD. Additionally, the GOLD ABE classification effectively predicted future events, even in dnsCOPD and PRISm patients.

These findings provide unique insight into the treatment and risk profiles of these previously overlooked patient groups within physician diagnosed COPD by using real-life data.

**Implications of all the available evidence**

Further research is crucial to assess the efficacy of current treatments, develop new approaches and bridge the knowledge gap regarding this clinically relevant patient population.

## Introduction

Chronic obstructive pulmonary disease (COPD) is currently defined as a heterogenous lung condition characterized by chronic respiratory symptoms (dyspnoea, cough, sputum production) and/or exacerbations.^1^ In the appropriate context, the presence of non-fully reversible airflow limitation measured by spirometry (forced expiratory volume in 1 s (FEV_1_)/forced vital capacity (FVC) < 0.7 post-bronchodilation) confirms the diagnosis. However, the Global Initiative for Chronic Obstructive Lung Disease (GOLD) document acknowledges that patients may present with structural lung lesions, e.g., emphysema or physiological abnormalities such as low-normal FEV_1_, lung hyperinflation, low diffusing capacity or accelerated lung function decline, without the presence of persistent airflow obstruction (FEV_1_/FVC ≥ 0.7 post-bronchodilation).^1^ GOLD proposes the term pre-COPD for these individuals. The term PRISm (Preserved Ratio Impaired Spirometry) has been introduced to define a group of patients with a normal FEV_1_/FVC ratio (≥0.7 post-bronchodilation) but abnormal FEV_1_ (<80% of predicted value).^1^^,^^2^ Patients with pre-COPD or PRISm are at risk of developing substantial persistent airflow obstruction over time, although not all individuals progress, and transitions between normal and obstructed spirometry can occur over time.^3^^,^^4^

The prevalence of pre-COPD and PRISm in population-based studies ranges from 7% to 20%, is higher with current and former smoking, and with both low and high body mass index (BMI), and the conditions are associated with increased mortality.^3^^,^^5^ Pre-COPD and PRISm are highlighted in the GOLD 2024 report as a group of patients of special interest, since they have been excluded from many studies and represent an important knowledge gap.^1^ Pre-COPD and PRISm have primarily been investigated in cohorts of (ex-) smokers like COPDGene,^2^ or population-based cohorts such as The Copenhagen City Heart Study^4^ or the Rotterdam study.^6^ However, no prior study has systematically assessed the risk of exacerbation, hospitalisation, or cause-specific mortality, in patients with physician diagnosed COPD (dCOPD) categorised into physician diagnosed COPD with normal spirometry (dnsCOPD) or PRISm and spirometrically confirmed COPD (sCOPD).

The Swedish National Airway Register (SNAR) continuously collects data on patients diagnosed with obstructive lung diseases and reflects the clinical practice related to COPD monitoring and management in both primary and secondary care in Sweden. We aimed to: first, identify and characterize patients who were spirometrically normal and PRISm within the cohort of physician-diagnosed COPD in SNAR; second, to stratify them further according to symptoms and exacerbations risk using the GOLD A/B/E classification; and third, to assess differences between dnsCOPD, PRISm, and spirometrically confirmed COPD patients in relation to future risk for exacerbations, and cause-specific hospitalisations and mortality.

## Methods

### Study population

In this nationwide cohort study, patients with data in SNAR from January 2014 until June 2022, 30 years or older at index date, with physician-diagnosed COPD (dCOPD) and a complete lung function test with post-bronchodilator values (FEV_1_, FVC, FEV_1_/FVC ratio), were included.^7^ Patients with a concomitant physician diagnosis of asthma recorded in SNAR were excluded. The index date was set as the date of the first recorded lung function test in SNAR within the predefined period. All COPD patients recorded in SNAR who met the predefined inclusion criteria were included, and the size of the cohort reflects the available data on patients in SNAR within the study period. The SNAR has been described previously,^7^ additional information and details regarding representativeness, data collection and changes to the register during the study period is available in the Online Supplementary.

Supplementary Fig. E1 provides an overview of the covariate and outcome assessment in the different registers. For covariates pertaining to exacerbations, cardiovascular comorbidities, pharmacological treatments and outcome variables, we used data linkage between the SNAR, the National Patient Register (NPR), the National Cause of Death Register (NCDR), and the National Prescribed Drug Register (NPDR) to obtain relevant data. The Swedish personal identity number, which means that each citizen is assigned a unique 10 digit number at birth, allows for complete linkage to other registers with very high accuracy.^8^

### Exposure groups

**Fig. 1:**
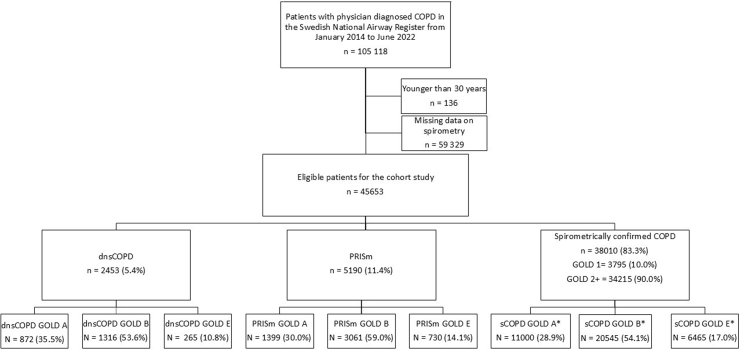
Flow diagram for eligible patient selection and distribution of physician diagnosed COPD (dCOPD) patients with dnsCOPD, PRISm and sCOPD. Abbreviations: PRISm = Preserved Ratio Impaired Spirometry, sCOPD = Spirometrically confirmed COPD, dnsCOPD = Physician diagnosed COPD with normal spirometry, GOLD 1 = normal FEV1, GOLD 2+ = FEV1 < 80% of predicted, ∗ Including GOLD 1 and GOLD 2+.

dCOPD patients who met the inclusion criteria were categorized into three groups based on post-bronchodilator spirometric findings: physician diagnosed COPD with normal spirometry (dnsCOPD) (FEV_1_/FVC ≥ 0.7 and FEV_1_ ≥ 80% of predicted), Preserved Ratio Impaired Spirometry (PRISm) (FEV_1_/FVC ≥ 0.7 and FEV_1_ < 80% of predicted), and spirometrically confirmed COPD (sCOPD) (FEV_1_/FVC <0.7) (Fig. 1). sCOPD patients were further divided into sCOPD GOLD stage 1 (FEV_1_ ≥ 80% of predicted) and sCOPD GOLD stage 2+ (FEV_1_ <80% of predicted) in some analyses. To calculate FEV_1_ percent of predicted, Swedish reference values by Hedenström et al. developed from pulmonary function tests on smoking and non-smoking adults in the Swedish general population were used.^1^^,^^9^^,^^10^ Patients in all three spirometric groups were also divided into GOLD groups A/B/E, based on symptom severity and exacerbation history for further analyses. Fig. 1 shows a flow diagram of the different groups.

To define patients’ baseline history of exacerbations, we used a previously validated method, also used in an earlier publication on exacerbation history and cardiovascular risk on SNAR data.^11^^,^^12^ Moderate exacerbations were defined as short-term oral corticosteroid prescriptions Anatomical Therapeutic Chemical Classification System (ATC) code H02AB for COPD, with dispensed prescription data retrieved from the NPDR.^13^ Severe exacerbations were defined as respiratory hospitalisations by the International Classification of Diseases, Tenth Revision.

(ICD-10) codes J41–J44, J96, J12–J18, J20–J22 from the NPR, listed as the main or secondary diagnosis.^14^ To define high vs. low symptom burden, patients were primarily categorized on the basis of the COPD assessment test (CAT) score cutoff of 10 points, or else an Modified Medical Research Council Dyspnoea scale (mMRC) score of 2 or higher was used. Patients with a CAT-score <10 points and no history of hospital admissions for exacerbation and fewer than two moderate exacerbations within 1 year before the index date were defined as GOLD A, patients with a CAT-score ≥10 points and no history of hospital admissions for exacerbation and fewer than two moderate exacerbations within 1 year before the index date as GOLD B, and patients with at least one hospital admission for exacerbation or two or more moderate exacerbations within 1 year before the index date was defined as GOLD E irrespective of their CAT-score.

### Outcomes

The primary outcomes included moderate exacerbations, as well as all-cause, respiratory, and cardiovascular hospitalisations and mortality. Patients were followed from the index date until the outcome event or death occurred or the end of follow-up on 31 November 2022. For moderate exacerbations, date of dispensation was obtained from the SPDR. For hospitalisations and deaths, ICD-10 codes J41–J44, J96, J12–J18, or J20–J22 were used for respiratory hospitalisations and I21 or I22, I61 or I63 for cardiovascular hospitalisation. These codes were recorded either as the primary diagnosis at hospital admission or as the underlying cause of death. Admission dates for hospitalisations were obtained from the NPR.^14^ Mortality data, including the date of death, was obtained from the NCDR.^15^ For all-cause hospitalisation the first hospitalisation after the index date, within the study period was used, irrespective of diagnosis code(s).

### Covariates

Clinical/demographic covariates were extracted from SNAR, in addition to spirometric values post bronchodilation (expressed numerically as litres), including symptom scores CAT score (ranging from 0 to 40, where higher scores indicate worse symptoms) and mMRC score (ranging from 0 to 4, with higher scores indicating more severe breathlessness), as well as age, height, sex (binary value (male/female) as recorded in The Swedish Tax Agency registers), smoking status and BMI (kg/m^2^), for which the latest registered datapoint in the period 18 months before the index date was used. Cardiovascular disease and other comorbidities at baseline were categorical yes/no and based on the presence or absence of predefined ICD-codes in the NPR in the 15 years before the index date (Supplementary Table E1). The use of inhaler therapy and cardiovascular pharmacological treatment was retrieved from the SPDR and defined as dispensations within 1 year before the index date (Supplementary Tables E2 and E3 for ATC codes and grouping). Smoking was categorized as current smoker, former smoker and never smoker, with former smokers defined as those who reported not smoking during the last 6 months at their latest reporting datapoint. BMI was categorized as normal weight (18.5–24.9 kg/m^2^), underweight (<18.5 kg/m^2^), overweight (25–29.9 kg/m^2^), and obese (≥30 kg/m^2^). Supplementary Fig. E1 illustrates the different lookback-periods for each of the covariates as well as the exacerbation history used for exposure groups GOLD A/B/E.

### Statistical analyses

Comparison of variables between eligible study participants and those excluded due to missing spirometry data was performed using the student t-test or chi-square test as appropriate and Mann–Whitney U-test for non-normally distributed data. Descriptive statistics were used to describe the baseline characteristics of the study participants. For continuous variables with a normal distribution, the mean ± standard deviation (SD) are reported. Categorical variables are presented as counts and percentages.

Competing risk regression, specifically the Fine–Gray model, was used to estimate the subdistribution hazard ratios (SHRs) of moderate exacerbations, hospitalisation and mortality, for different spirometric groups of dCOPD patients as the main study exposure.^16^ This model is commonly used in survival analysis to estimate risk by calculating SHRs and cumulative incidence functions for a specific event, whilst taking into account the presence of competing risks, such as death from other causes, to provide more accurate estimates.

**Fig. 2:**
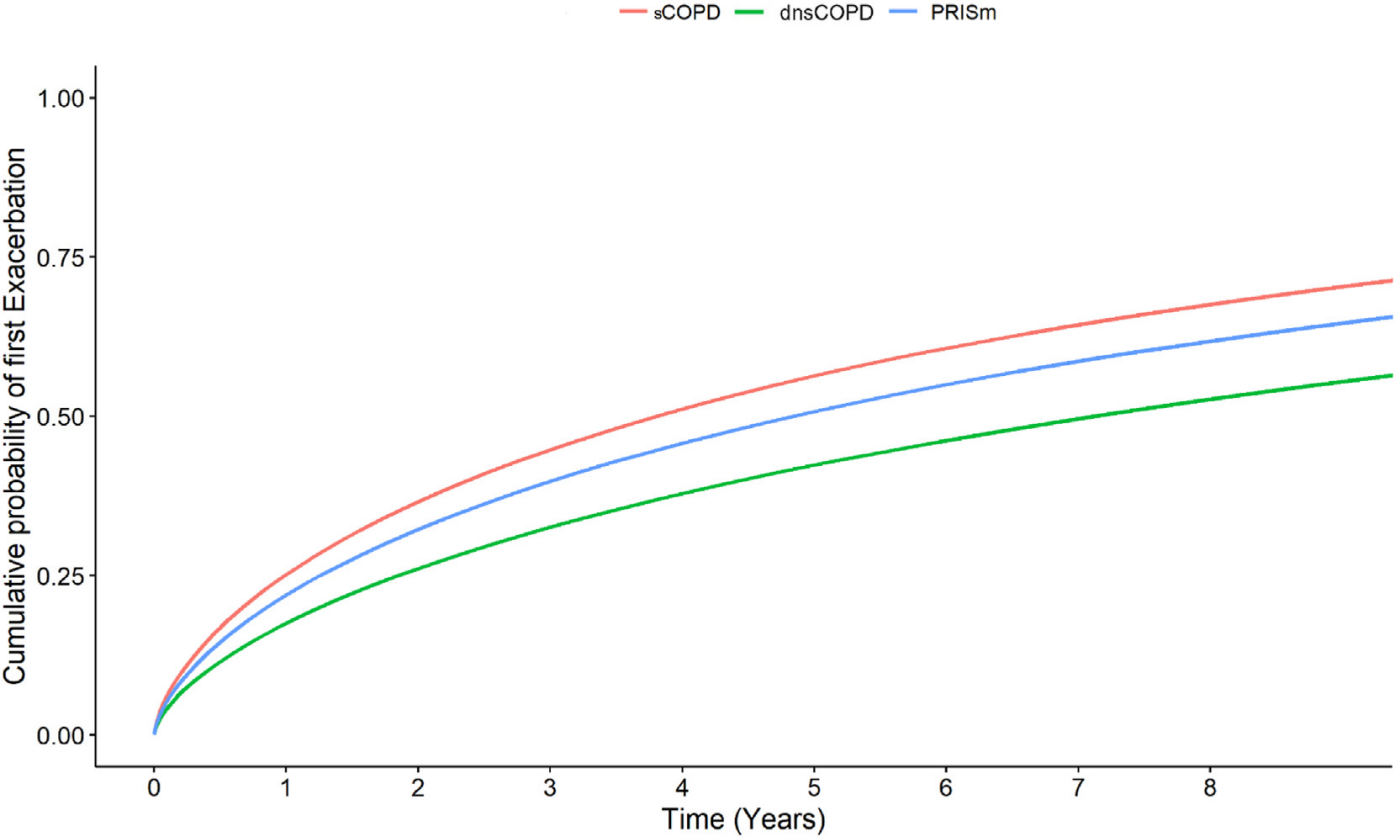
Cumulative probability of first exacerbation in physician diagnosed COPD (dCOPD) in patients with dnsCOPD, PRISm and sCOPD.

**Fig. 3:**
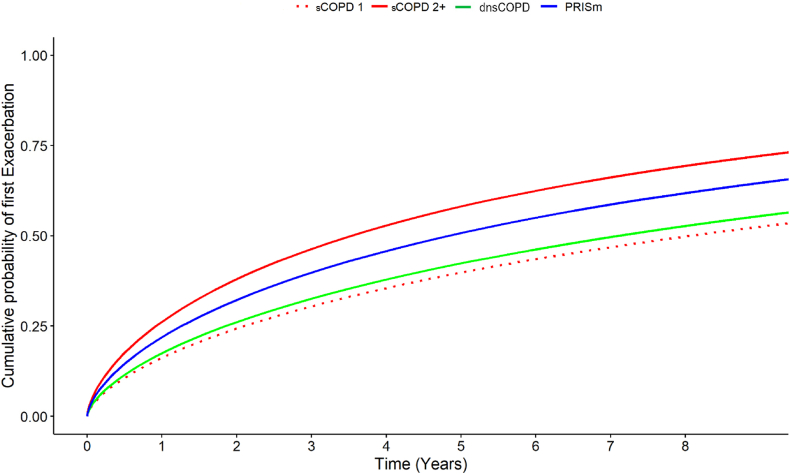
Cumulative probability of first exacerbation in physician diagnosed COPD (dCOPD) in patients with dnsCOPD, PRISm, sCOPD stage 1 and sCOPD stages 2,3,4.

**Fig. 4:**
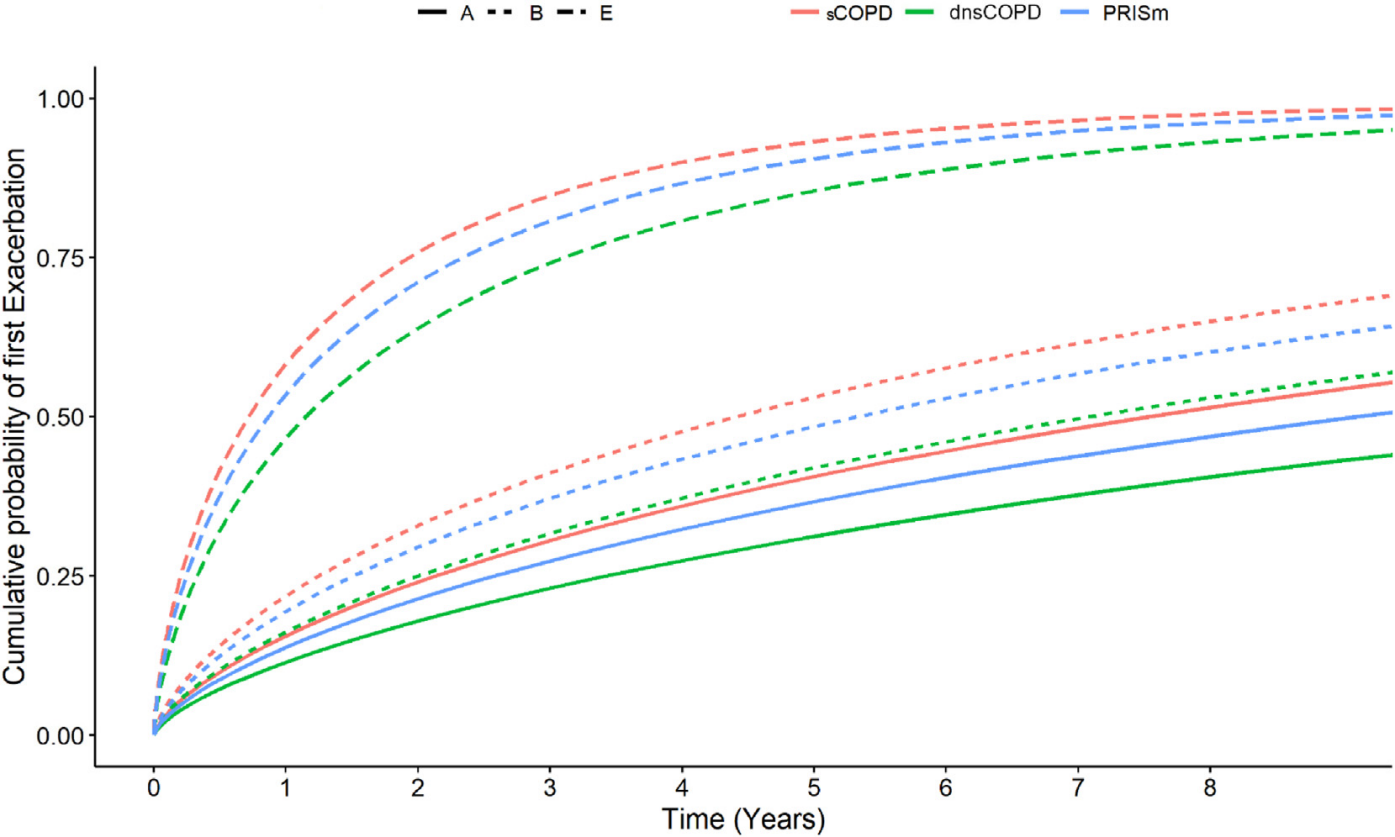
Cumulative probability of first exacerbation in physician diagnosed COPD (dCOPD) in patients with dnsCOPD, PRISm, sCOPD further categorized into groups A, B and E.

In the primary analysis of dCOPD, dnsCOPD and PRISm patients were compared with sCOPD patients as the reference. In a secondary analysis, GOLD stage 1 sCOPD patients we compared to patients dnsCOPD, and sCOPD GOLD stage 2+ (stages 2, 3 and 4) to PRISm, as done in a previous population-based cohort study.^6^ In a third analysis, we categorized dnsCOPD, PRISm and sCOPD into GOLD groups A, B and E, respectively, and compared these nine groups, with sCOPD E as baseline. Cumulative probability of distribution curves for the different outcomes according to our 3 different categorization approaches were produced to accompany the SHRs, in line with the recommendations for reporting results from competing risk regression (Fig. 2, Fig. 3, Fig. 4, Supplementary Figs. E2–E7 panels A–C).^16^

For all 3 different analysis comparisons, and for all outcomes except all-cause mortality, crude and adjusted subdistribution hazard ratios (SHRs) with 95% confidence intervals (CIs) were estimated, considering death from other causes as a competing risk.^16^^,^^17^ For the outcome of all-cause mortality, a Cox proportional hazards model was used to computed crude and adjusted hazard ratios (HRs) with 95% CIs. Covariates used in all of the adjusted models including all adjusted competing risk regression models and cox-proportional hazard models were age, sex, BMI, smoking status, baseline cardiovascular disease and baseline cardioprotective medications.^18^ We defined statistical significance as 95% confidence intervals (CIs) for SHRs and HRs not including 1.0. This criterion was used to assess the robustness of associations.

We conducted sensitivity mediation analyses to examine whether baseline cardiovascular comorbidities and cardioprotective medications mediated the association between spirometric categories (PRISm, dnsCOPD, and sCOPD) and the outcomes; exacerbations, respiratory mortality, cardiovascular mortality, and all-cause mortality. Mediation effects were assessed using the average causal mediation effect (ACME), average direct effect (ADE), and proportion mediated (Supplementary Tables E8 and E9).

To handle missing data on the covariates BMI (2.5%) and smoking (5.2%), we treated missing as a factor level, in order to avoid selection bias affecting the results. This means that the same cohort of patients was analysed in the crude and adjusted models.

The baseline data were defined using SAS (version 15.2), and R (version 4.3.1) was used for further data management. The package cmprsk (version 2.2–11) was used for the Fine–Gray competing risk regression analyses and the package survival (version 3.7–0) was used for Cox proportional hazards regression models.

### Ethical approval

This study was approved by the Swedish Ethical Review Authority (2019–04915). Following a physician’s diagnosis of COPD and obtaining informed consent, registrations of patient data are directly transferred from electronic medical records into the SNAR. Patients can choose to opt out and have their data deleted at any point in time.

### Role of the funding source

The funding sources had no role in study design, data collection, data analysis, data interpretation, or writing of the report. The first author and the corresponding author had full access to all the data and had final responsibility for the decision to submit for publication.

## Results

### Study population and characteristics

Of 105,118 identified patients with dCOPD, 45,653 patients (43.4%) were eligible for the current study (Fig. 1). Patients excluded because of missing spirometry data compared to those included had similar sex distribution (43.5% vs. 43.7% males, p = 0.68), had a slightly higher mean age (71.2 ± 10.2 vs. 70.8 years ± 9.1, p < 0.001), mean BMI (27.6 ± 14.8 vs. 26.8 ± 5.7 kg/m^2^, p < 0.001), and a higher proportion of never smokers (11.7% vs. 9.4%, p < 0.001).

Eligible patients were classified into dnsCOPD (5.4%), PRISm (11.4%) or sCOPD (83.3% (stage 1: 10.0%, stage 2+: 90.0%)). The distribution of groups A, B and E were - dnsCOPD: 35%, 54%, 11%; PRISm: 27%, 59%, 14%; and sCOPD: 29%, 54%, 17%, respectively (Fig. 1).

Table 1 shows the characteristics of all patients with dCOPD, also stratified for dnsCOPD, PRISm and sCOPD. The overall study population consisted mainly of elderly patients with moderate airflow limitation, 29.4% percent experienced at least one exacerbation in the last year and 7.4% had been hospitalised for COPD in the last year. More than half were former smokers and over one third were current smokers. Patients had an average BMI of 26.8 kg/m^2^. An overwhelming majority (80%) were treated with inhaler therapy, with 62.6% treated with an inhaled corticosteroid-containing combination.

**Table 1:** Baseline characteristics among 45,653 patients aged > 30 with complete spirometry and a physician diagnosis of COPD (dCOPD) registered in the Swedish National Airway Register, from 2014 to 2022, overall and by dnsCOPD, PRISm and sCOPD, as well as GOLD stage 1 vs. 2+.

	Total study population	dnsCOPD	PRISm	sCOPD	sCOPD GOLD stage 1	sCOPD GOLD stage 2+
Patients, n (%)	45,653	2453 (5.4)	5190 (11.4)	38,010 (83.3)	3795 (8.3)	34,215 (74.9)
Male sex, n (%)	19,934 (43.7)	1096 (44.7)	2208 (42.5)	16,630 (43.8)	1804 (47.5)	14,826 (43.3)
Age, years, mean (SD)	70.8 (9.1)	69.6 (10.3)	69.2 (9.3)	71.1 (8.9)	71.1 (9.7)	71.1 (8.8)
BMI, kg/m2 (n = 44,916), mean (SD)	26.8 (5.7)	27.9 (5.4)	29.6 (6.0)	26.4 (5.5)	25.8 (4.8)	26.4 (5.6)
**BMI category, n (%), (n** = **44,946)**						
Obese	11,200 (24.5)	735 (30.0)	2233 (43.0)	8232 (21.7)	595 (15.7)	7637 (22.3)
Overweight	15,397 (33.7)	938 (38.2)	1753 (33.8)	12,706 (33.4)	1381 (36.4)	11,325 (33.1)
Normal weight	15,889 (34.8)	688 (28.0)	1018 (19.6)	14,183 (37.3)	1623 (42.8)	12,560 (36.7)
Underweight	2460 (5.4)	59 (2.4)	106 (2.0)	2295 (6.0)	134 (3.5)	2161 (6.3)
**Smoking status, n (%), (n** = **43,274)**						
Current smoker	16,802 (36.8)	726 (29.6)	1814 (35.0)	14,262 (37.5)	1380 (36.4)	12,882 (37.7)
Former smoker	22,158 (48.5)	1209 (49.3)	2455 (47.3)	18,494 (48.7)	1754 (46.2)	16,740 (48.9)
Never smoker	4314 (9.4)	364 (14.8)	651 (12.5)	3299 (8.7)	460 (12.1)	2839 (8.3)
**Spirometry values**^a^**, mean (SD)**						
FEV_1_, liter	1.6 (0.7)	2.5 (0.6)	1.8 (0.5)	1.6 (0.6)	2.4 (0.6)	1.5 (0.6)
FEV_1_, % predicted	59.6 (18.1)	90.2 (9.0)	64.9 (10.8)	56.9 (17.4)	87.4 (6.8)	53.5 (14.7)
FVC, liter	2.8 (0.9)	3.3 (0.8)	2.4 (0.8)	2.8 (0.9)	3.8 (0.9)	2.7 (0.9)
FVC, % predicted	75.8 (16.9)	88.7 (10.3)	65.1 (12.0)	76.4 (17.0)	101.0 (10.0)	73.7 (15.4)
FEV_1_/FVC	0.6 (0.1)	0.8 (0.1)	0.8 (0.1)	0.6 (0.1)	0.6 (0.1)	0.5 (0.1)
**FEV_1_% predicted categories**^a^**, n (%)**						
FEV_1_ ≥ 80% predicted	6248 (13.7)	2453 (100.0)	NA	3795 (10.0)	3795 (100.0)	NA
50% ≤ FEV_1_<80% predicted	25,894 (56.7)	NA	4707 (90.7)	21,187 (55.7)	NA	21,187 (61.9)
30% ≤ FEV_1_<50% predicted	11,216 (24.6)	NA	450 (8.7)	10,766 (28.3)	NA	10,766 (31.5)
FEV_1_ < 30% predicted	2295 (5.0)	NA	33 (0.6)	2262 (6.0)	NA	2262 (6.6)
**Symptomatic burden, mean (SD)**						
CAT score (n = 42,457)	13.3 (7.0)	12.5 (6.7)	13.8 (7.1)	13.3 (7.1)	10.8 (5.9)	13.5 (7.1)
mMRC score (n = 27,756)	1.6 (1.1)	1.2 (0.9)	1.6 (1.1)	1.6 (1.2)	1.1 (0.9)	1.7 (1.2)
**ABE groups, n (%)**						
Group A	13,271 (29.1)	872 (35.5)	1399 (27.0)	11,000 (28.9)	1664 (43.8)	9336 (27.3)
Group B	24,922 (54.6)	1316 (53.6)	3061 (59.0)	20,545 (54.1)	1811 (47.7)	18,734 (54.8)
Group E	7460 (16.3)	265 (10.8)	730 (14.1)	6465 (17.0)	320 (8.4)	6145 (18.0)
**Exacerbation history, n (%)**						
0 Exacerbations	32,214 (70.6)	1856 (75.7)	3765 (72.5)	26,593 (70.0)	3105 (81.8)	23,488 (68.6)
1 moderate 0 severe	5979 (13.1)	332 (13.5)	695 (13.4)	4952 (13.0)	370 (9.7)	4582 (13.4)
>2 moderate 0 severe	4071 (8.9)	184 (7.5)	457 (8.8)	3430 (9.0)	202 (5.3)	3228 (9.4)
1 severe	2761 (6.0)	68 (2.8)	239 (4.6)	2454 (6.5)	107 (2.8)	2347 (6.9)
2 severe	467 (1.0)	8 (0.3)	26 (0.5)	433 (1.1)	6 (0.2)	427 (1.2)
3+ severe	161 (0.4)	5 (0.2)	8 (0.2)	148 (0.4)	5 (0.1)	143 (0.4)
**Inhalation treatment, n (%)**						
ICS	1599 (3.5)	104 (4.2)	221 (4.3)	1274 (3.4)	181 (4.8)	1093 (3.2)
LABA	925 (2.0)	44 (1.8)	104 (2.0)	777 (2.0)	79 (2.1)	698 (2.0)
LAMA	6391 (14.0)	434 (17.7)	855 (16.5)	5102 (13.4)	608 (16.0)	4494 (13.1)
SABA or SAMA	1775 (3.9)	118 (4.8)	232 (4.5)	1425 (3.7)	196 (5.2)	1229 (3.6)
ICS/LABA	4905 (10.7)	318 (13.0)	621 (12.0)	3966 (10.4)	437 (11.5)	3529 (10.3)
ICS/LABA/LAMA	15,118 (33.1)	535 (21.8)	1434 (27.6)	13,149 (34.6)	614 (16.2)	12,535 (36.6)
ICS/LAMA	1238 (2.7)	83 (3.4)	197 (3.8)	958 (2.5)	86 (2.3)	872 (2.5)
LABA/LAMA	4574 (10.0)	204 (8.3)	480 (9.2)	3890 (10.2)	274 (7.2)	3616 (10.6)
No inhalation treatment	9128 (20.0)	613 (25.0)	1046 (20.2)	7469 (19.7)	1320 (34.8)	6149 (18.0)

BMI: body mass index; FEV_1_: forced expiratory volume in the first second; FVC: forced vital capacity, NA = not applicable, CAT: COPD Assessment Test score (ranging from 0 to 40, where higher scores indicate worse symptoms), mMRC: modified Medical Research Council dyspnea score (ranging from from 0 to 4, with higher scores indicating more severe breathlessness), ICS: inhaled corticosteroids; LABA: long-acting beta-2 agonist; LAMA: long-acting muscarin antagonist; SABA: short-acting beta-2 agonist; SAMA: short-acting muscarin antagonist; ACE: Angiontensin-converting enzyme inhibitor; ARB: Angiotensin-receptor blocker.

a :All spirometric measurements were post-bronchodilation.

Patients with dnsCOPD and PRISm had a similar smoking history as sCOPD patients, with a majority of ex- or current smokers. Mean CAT scores were slightly lower in dnsCOPD compared to PRISm and sCOPD, but similar in PRISm and sCOPD. Mean CAT scores in dnsCOPD were higher compared to patients with sCOPD GOLD stage 1. The proportion of patients with a history of frequent moderate exacerbations was similar in sCOPD and PRISm, but severe exacerbations (hospitalisation) were more common in sCOPD. The large majority of dnsCOPD and PRISm patients were treated with inhaled therapy, with 22% and 28% respectively receiving triple inhaled therapy compared to 35% in sCOPD. A larger proportion of sCOPD GOLD stage 1 patients did not receive treatment (34.8%) compared to dnsCOPD (25.0%) and PRISm (20.2%). Patients with dnsCOPD and PRISm had a higher prevalence of overweight and obesity compared to sCOPD. Patients with PRISm had the highest prevalence of cardiovascular disease, particularly heart failure and hypertension and a higher prevalence of diabetes (Table 2).

**Table 2:** Baseline comorbidities and cardiovascular medications among 45,653 patients aged > 30 with complete spirometry and a physician diagnosis of COPD (dCOPD) registered in the Swedish National Airway Register, from 2014 to 2022, overall and by dnsCOPD, PRISm and sCOPD, as well as GOLD stage 1 vs. 2+.

	Total study population	dnsCOPD	PRISm	sCOPD	sCOPD GOLD stage 1	sCOPD GOLD stage 2+
Patients, n (%)	45,653	2453 (5.4)	5190 (11.4)	38,010 (83.3)	3795 (8.3)	34,215 (74.9)
**Baseline comorbidities, n (%)**						
Myocardial infarction	1625 (3.6)	89 (3.6)	200 (3.9)	1336 (3.5)	124 (3.3)	1212 (3.5)
Stroke	1290 (2.8)	70 (2.9)	154 (3.0)	1066 (2.8)	94 (2.5)	972 (2.8)
Heart failure	3920 (8.6)	152 (6.2)	546 (10.5)	3222 (8.5)	168 (4.4)	3054 (8.9)
Atrial fibrillation	5174 (11.3)	218 (8.9)	715 (13.8)	4241 (11.2)	301 (7.9)	3940 (11.5)
Coronary heart disease	5758 (12.6)	308 (12.6)	724 (13.9)	4726 (12.4)	421 (11.1)	4305 (12.6)
Hypertension	14,715 (32.2)	762 (31.1)	1941 (37.4)	12,012 (31.6)	982 (25.9)	11,030 (32.2)
Peripheral artery disease	1604 (3.5)	58 (2.4)	169 (3.3)	1377 (3.6)	98 (2.6)	1279 (3.7)
Diabetes mellitus type 2	4564 (10.0)	250 (10.2)	865 (16.7)	3449 (9.1)	209 (5.5)	3240 (9.5)
Deep vein thrombosis	838 (1.8)	50 (2.0)	113 (2.2)	675 (1.8)	75 (2.0)	600 (1.8)
Pulmonary Embolism	656 (1.4)	32 (1.3)	62 (1.2)	562 (1.5)	43 (1.1)	519 (1.5)
Any cardiovascular disease	19,000 (41.6)	948 (38.6)	2496 (48.1)	15,556 (40.9)	1276 (33.6)	14,280 (41.7)
**Cardiovascular treatment, n (%)**						
Anticoagulation	18,193 (39.9)	900 (36.7)	2254 (43.4)	15,039 (39.6)	1329 (35.0)	13,710 (40.1)
Anti-arrhythmic	5089 (11.1)	302 (12.3)	666 (12.8)	4121 (10.8)	354 (9.3)	3767 (11.0)
Diuretics	535 (1.2)	21 (0.9)	72 (1.4)	442 (1.2)	29 (0.8)	413 (1.2)
Betablocker	11,225 (24.6)	485 (19.8)	1551 (29.9)	9189 (24.2)	618 (16.3)	8571 (25.1)
Calcium channel-blocker	16,286 (35.7)	778 (31.7)	2179 (42.0)	13,329 (35.1)	1063 (28.0)	12,266 (35.8)
ACE/ARB	12,080 (26.5)	606 (24.7)	1449 (27.9)	10,025 (26.4)	890 (23.5)	9135 (26.7)
Statin	20,928 (45.8)	1094 (44.6)	2700 (52.0)	17,134 (45.1)	1537 (40.5)	15,597 (45.6)
Diabetes	17,175 (37.6)	941 (38.4)	2252 (43.4)	13,982 (36.8)	1298 (34.2)	12,684 (37.1)
Any cardiac drug	6274 (13.7)	358 (14.6)	1121 (21.6)	4795 (12.6)	319 (8.4)	4476 (13.1)
Any metabolic drug	32,566 (71.3)	1688 (68.8)	3934 (75.8)	26,944 (70.9)	2446 (64.5)	24,498 (71.6)
Any cardiometabolic drug	18,764 (41.1)	1038 (42.3)	2507 (48.3)	15,219 (40.0)	1396 (36.8)	13,823 (40.4)

### Outcomes

Follow-up extended for a median of 3.74 years (IQR 2.15–5.04) for all-cause mortality (163,215 person-years), 1.96 years (IQR 0.81–3.75) for all-cause hospitalisation, and 1.90 years (IQR 0.69–3.83) for moderate exacerbations (Table 3). The exacerbation rate for dnsCOPD patients was 0.13 events per patient-year, for PRISm 0.17 and 0.20 for sCOPD (Table 3).

**Table 3:** Follow up times and incidence rates for a first exacerbation, first hospitalisation, and mortality, among 45,653 patients aged >30 with complete spirometry and a physician diagnosis of COPD (dCOPD) registered in the Swedish National Airway Register, overall and by dnsCOPD, PRISm and sCOPD as well as sCOPD in GOLD stage 1 and 2+.

	Total study population	dnsCOPD	PRISm	sCOPD	sCOPD GOLD stage 1	sCOPD GOLD stage 2+
Patients, n (%)	45,653	2453 (5.4)	5190 (11.4)	38,010 (83.3)	3795 (8.3)	34,215 (74.9)
**Exacerbations**						
Median follow-up time, years (IQR)	1.90 (0.69–3.83)	2.34 (0.85–4.09)	2.09 (0.76–3.93)	1.86 (0.67–3.80)	2.70 (1.00–4.13)	1.79 (0.64–3.76)
Follow-up time exacerbations person-years	107,009	6331	12,683	87,996	10,224	77,772
Exacerbations, n (%)	20,838 (100)	850 (34.7)	2179 (42.0)	17,809 (46.9)	1227 (32.3)	16,582 (48.5)
Exacerbation incidence, (events/patient-year)	0.19	0.13	0.17	0.20	0.12	0.21
**Hospitalisation**						
Median follow-up time, all-cause hospitalisation, years	1.96 (0.81–3.75)	2.15 (0.93–3.80)	1.96 (0.81–3.74)	1.95 (0.81–3.75)	2.28 (0.95–3.91)	1.92 (0.79–3.73)
Follow-up time all-cause hospitalisations person-years	108,099	6027	12,218	89,854	9665	80,189
All-cause hospitalisations, n (%)	23,890	1092 (44.5)	2653 (51.1)	20,145 (53.0)	1570 (41.4%)	18,575 (54.3)
All-cause hospitalisation incidence, (events/patient-year)	0.22	0.18	0.22	0.22	0.16	0.23
Respiratory hospitalisations, n (%)	8084	173 (7.1)	630 (12.1)	7281 (19.2)	237 (6.2)	7044 (20.6)
Follow-up time respiratory hospitalisations person-years	146,889	8194	17,147	121,548	12,901	108,647
Respiratory hospitalisation incidence, (events/patient-year)	0.06	0.02	0.04	0.06	0.02	0.06
Cardiovascular hospitalisations, n (%)	2331	97 (4.0)	264 (5.1)	1970 (5.2)	157 (4.1)	1813 (5.3)
Follow-up time cardiovascular hospitalisations person-years	158,842	8329	17,946	132,568	13,025	119,542
Cardiovascular hospitalisation incidence, (events/patient-year)	0.01	0.01	0.01	0.01	0.01	0.02
**Mortality**						
Median follow-up time, all-cause mortality, years	3.74 (2.15–5.04)	3.61 (2.05–4.88)	3.71 (2.15–5.03)	3.76 (2.15–5.05)	3.67 (2.13–4.91)	3.76 (2.15–5.06)
Follow-up time all-cause mortality person-years	163,215	8510	18,453	136,251	13,331	122,920
All-cause deaths	7744	214	722	6808	332	6476
All cause deaths, events/patient year	0.05	0.03	0.04	0.05	0.02	0.05
Respiratory deaths	1429	14	79	1336	24	1312
Respiratory deaths, events/patient year	0.01	0.00	0.00	0.01	0.00	0.01
Cardiovascular deaths	340	7	27	306	25	281
Cardiovascular deaths, events/patient year	0.00	0.00	0.00	0.00	0.00	0.00

**Fig. 5:**
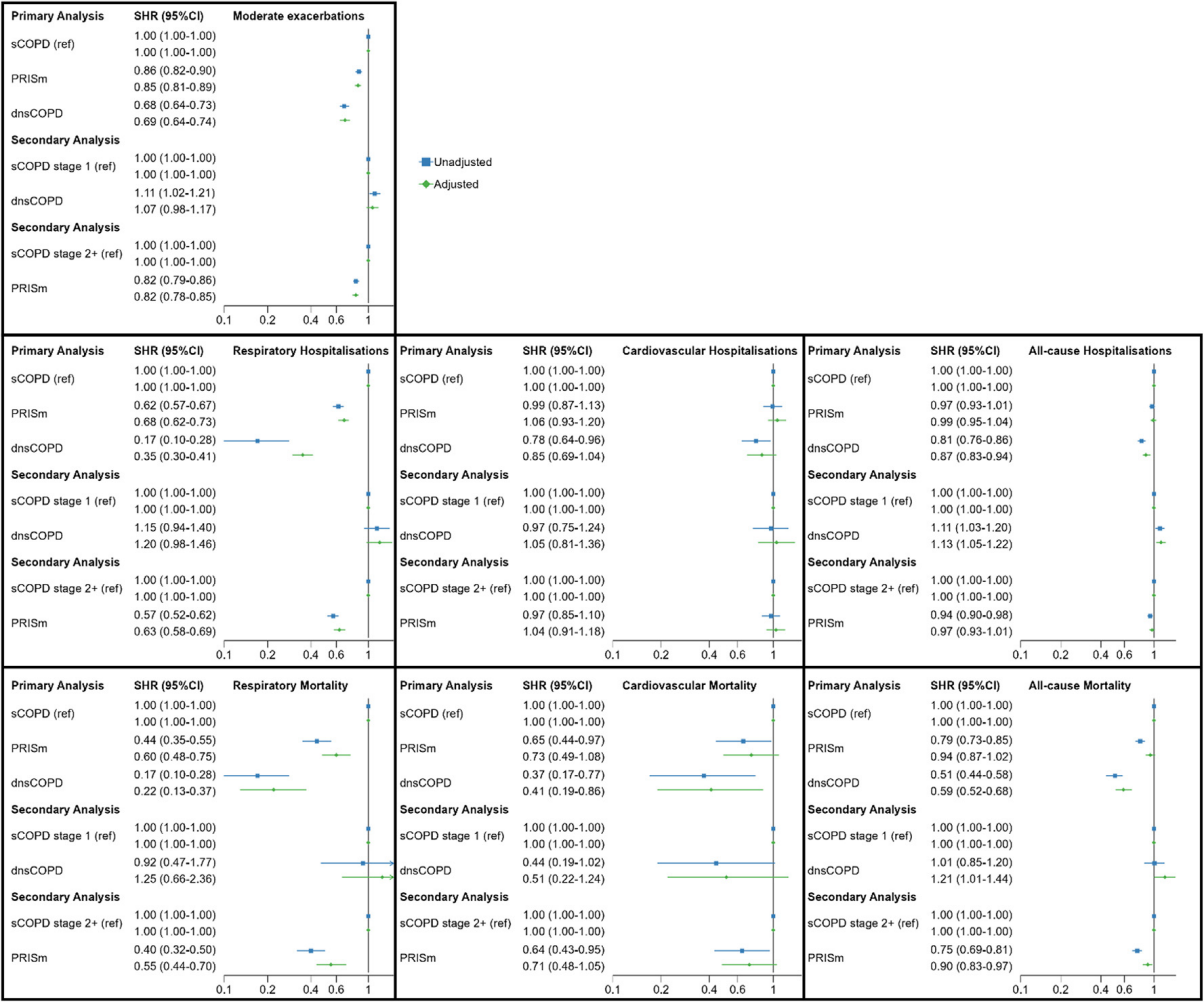
Crude and adjusted subdistribution hazard ratios for a first event of moderate exacerbation, respiratory, cardiovascular and all-cause- hospitalisation and mortality among 45,653 patients aged > 30 with complete spirometry and a physician diagnosis of COPD (dCOPD) registered in the Swedish National Airway Register, from 2014 to 2022. Primary analysis: dnsCOPD and PRISm compared to sCOPD as reference. Secondary analysis: dnsCOPD compared to sCOPD stage 1 as reference & PRISm compared to sCOPD stages 2, 3 and 4 as reference. Notes: SHR: Subdistribution Hazard Ratio; CI: confidence interval; HR: Hazard Ratio, dnsCOPD = physician diagnosed COPD with normal spirometry, PRISm = Preserved Ratio Impaired Spirometry, sCOPD = Spirometrically confirmed COPD, Adj. = Adjustment for age, sex, BMI, smoking status, previous cardiovascular disease and previous cardioprotective medications; ∗ Death from another cause was considered as a competing risk.

DnsCOPD patients had a significantly and substantially lower risk of future exacerbation SHR 0.69 95%CI (0.64–0.74), all-cause hospitalisations SHR 0.87 95%CI (0.83–0.94) and respiratory hospitalisations SHR 0.40 95%CI (0.34–0.46), as well as all-cause mortality HR 0.59 95%CI (0.52–0.68), cardiovascular mortality SHR 0.41 95%CI (0.19–0.86) and respiratory mortality SHR 0.22 95%CI (0.13–0.37), than sCOPD patients (Fig. 5, Supplementary Figs. E2–E7 panels A). All-cause hospitalisation risk was lower in dnsCOPD SHR 0.87 95% CI (0.83–0.94), but similar in PRISm SHR 0.99 95%CI (0.95–1.04) compared to sCOPD. Cardiovascular hospitalisation in dnsCOPD SHR 0.85 95%CI (0.69–1.04) and PRISm SHR 1.06 95%CI (0.93–1.20) was similar compared to sCOPD (Fig. 5). Cardiovascular mortality SHR 0.73 95%CI (0.49–1.08) and all-cause mortality HR 0.94 95%CI (0.87–1.02) in PRISm was comparable to COPD, while it was lower in dnsCOPD SHR 0.41 95%CI (0.19–0.86) and HR 0.59 95%CI (0.52–0.68), respectively, compared to sCOPD (Fig. 5, Supplementary Figs. E4 and E6 panels A).

DnsCOPD compared to sCOPD GOLD stage 1 patients had a higher risk of all-cause hospitalisation SHR 1.13 95%CI (1.05–1.22) and all-cause mortality HR 1.21 95%CI (1.01-1.44), but a similar non-significantly different risk for exacerbations SHR 1.07 95%CI (0.98–1.17) and respiratory hospitalisation SHR 1.25 95%CI (0.66–2.36) or cardiovascular hospitalisation SHR 1.05 95%CI (0.81–1.36) or cardiovascular mortality SHR 0.51 95%CI (0.22–1.24) (Fig. 5 and Supplementary Figs. E2–E7 panels B). PRISm compared to sCOPD GOLD stage 2+ had a lower risk of exacerbations SHR 0.82 95%CI (0.78–0.85), respiratory hospitalisation 0.63 95%CI (0.58–0.69), respiratory mortality SHR 0.55 95%CI (0.44–0.70) and all-cause mortality HR 0.55 95%CI (0.44–0.70) but a similar risk for all-cause hospitalisation SHR 0.97 95%CI (0.93–1.01) and cardiovascular hospitalisation SHR 1.04 95%CI (0.91–1.18) and cardiovascular mortality SHR 0.71 95%CI (0.48–1.05) (Fig. 5 and Supplementary Figs. E2–E7 panels B).

In the analysis substratified by GOLD A/B/E, dnsCOPD and PRISm group E patients had a higher risk for all outcomes, compared to sCOPD group B patients. Similarly, dnsCOPD and PRISm group B patients demonstrated a higher risk compared to sCOPD group A patients (Fig. 4, Fig. 6, and Supplementary Figs. E2–E7 panels C).

**Fig. 6:**
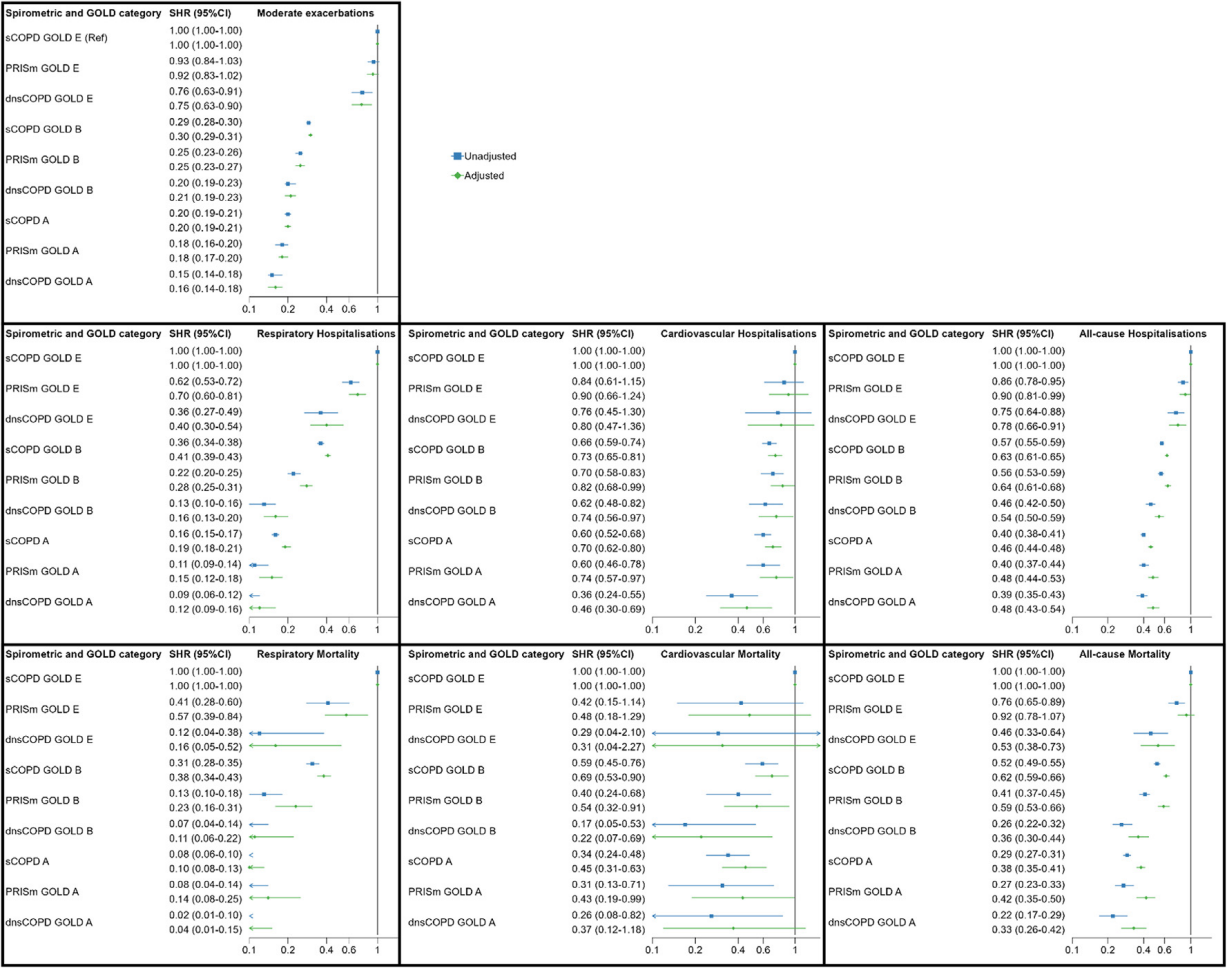
Crude and adjusted subdistribution hazard ratios for a first event of moderate exacerbation, respiratory, cardiovascular and all-cause- hospitalisation and mortality among 45,653 patients aged > 30 with complete spirometry and a physician diagnosis of COPD (dCOPD) registered in the Swedish National Airway Register, from 2014 to 2022 by dnsCOPD, PRISm and sCOPD further stratified according to A, B, E. sCOPD GOLD E as Reference. Notes: SHR: Subdistribution Hazard Ratio; CI: confidence interval; HR: Hazard Ratio, dnsCOPD = physician diagnosed COPD with normal spirometry, PRISm = Preserved Ratio Impaired Spirometry, sCOPD = Spirometrically confirmed COPD, Adj. = Adjustment for age, sex, BMI, smoking status, previous cardiovascular disease and previous cardioprotective medications; ∗Death from another cause was considered as a competing risk.

The mediation analyses showed that cardiovascular comorbidities and medications had statistically significant mediation effects on the main outcomes, however the proportion of mediated effect was small across all outcomes (<10%) (Supplementary Tables E8 and E9).

## Discussion

This is the first large-scale study to identify and clinically characterize patients with a physician diagnosed COPD but normal spirometry (dnsCOPD) and PRISm in a real-world cohort of patients with physician-diagnosed COPD, and evaluated their risk of exacerbations, as well as overall and cause-specific hospital admissions and mortality. Our study provides a unique perspective on PRISm and dnsCOPD based on physician-diagnosed cases in register-based real-world clinical data. DnsCOPD and PRISm patients were symptomatic, experienced exacerbations and were often prescribed inhaled therapy, similar to patients with spirometrically confirmed COPD. Notably, PRISm patients had an increased prevalence of obesity, diabetes, and cardiovascular disease, while dnsCOPD and PRISm generally had lower risk of exacerbations, respiratory hospitalisations and respiratory mortality than spirometrically confirmed COPD patients. The rate of hospitalisation for cardiovascular and any cause was similar in PRISm patients compared to spirometrically confirmed COPD patients. The GOLD A/B/E- classification, based on symptoms and exacerbation history, was predictive for future exacerbations, even in patients with dnsCOPD or PRISm. Indeed, the A/B/E classifications seemed to have a stronger association with the main outcomes, compared to stratification into dnsCOPD, PRISm or spirometrically confirmed COPD.

Previous research has established a link between PRISm and a range of disease outcomes, including diabetes, metabolic syndrome, hypertension, stroke and cardiovascular disease.^19^, ^20^, ^21^ These findings are reinforced by the current analyses, which show that patients with this spirometric phenotype of physician-diagnosed COPD have a very high prevalence of obesity, and are more likely to suffer from cardiovascular disease and diabetes. The proportion of current and former smokers were similar in both dnsCOPD and PRISm, and similar to spirometrically confirmed COPD. A previous study found that smoking prevalence was remarkably high in trajectories related to PRISm.^4^ This suggests that smoking, in addition to causing airflow limitation, could also be linked to restrictive lung function pattern, possibly in conjunction with other factors such as overweight or excess adiposity.

Overall, among all patients with physician diagnosed COPD, patients with dnsCOPD and PRISm resemble to some extent those with spirometrically confirmed COPD in terms of smoking history, symptom severity, and exacerbation history. Moreover, physicians evidently prescribe COPD-related inhalation therapy to those with dnsCOPD and PRISm, at rates comparable to patients with spirometrically confirmed COPD. This aligns with findings from the COPDGene cohort, indicating that many smokers without airflow-limitation still receive COPD medications and experience COPD-like symptoms and exacerbation-like events.^22^^,^^23^ The efficacy of this treatment approach remains to be proven. A recent study demonstrated that inhaled dual bronchodilator therapy did not reduce respiratory symptoms in symptomatic, tobacco-exposed persons with preserved lung function.^24^ It is important to note that this study did not examine the use of inhaled corticosteroids (ICS) nor the impact of bronchodilator therapy, with or without ICS, on exacerbations or other outcomes.

Although lower compared to spirometrically confirmed COPD, we observed a relatively high risk for future COPD-exacerbations as well as respiratory admissions, in both dnsCOPD and PRISm. Previous studies have similarly found a risk for respiratory and COPD-related admissions in patients with PRISm.^4^ The risk of exacerbations and respiratory admissions increases progressively from dnsCOPD to PRISm to spirometrically confirmed COPD. The A/B/E classification, which is based on symptoms and exacerbations, was more strongly associated with our main outcomes, compared to spirometric categorization into dnsCOPD, PRISm and spirometrically confirmed COPD.

Interestingly, when comparing the relative risk of sCOPD GOLD stage 1 patients to dnsCOPD, the risk were similar for almost all outcomes, with a small but significantly increased risk for all-cause hospitalisation for the dnsCOPD group compared to sCOPD GOLD stage 1 patients. This may be attributable to the increased symptomatic burden and exacerbation history of the dnsCOPD group compared to sCOPD GOLD stage 1, which are key drivers of hospitalisations.^25^ This suggests that dnsCOPD represents an important clinical group of patients with elevated risks that are not captured by spirometry alone. Possibly, an assessment based on symptoms and exacerbation history in patients presenting with a clinical picture of COPD can better identify patients who might benefit from early interventions regardless of whether they meet the strict spirometric criteria for COPD.

The question remains whether patients diagnosed with physician diagnosed COPD with FEV_1_/FVC ≥0.70 (i.e., dnsCOPD or PRISm) should be considered misdiagnosed. Epidemiological studies have consistently shown that a large proportion of patients with physician-diagnosed COPD exhibit unobstructed post-bronchodilator spirometry, raising concerns about overdiagnosis.^26^^,^^27^ This was also recently seen in a report of the Novelty study, a clinical cohort of patients with physician diagnosis of COPD.^23^ However, in clinical practice, as shown in the current study, these patients are typically elderly, smokers or former smokers, with chronic respiratory symptoms (especially dyspnoea), who might seek medical help because of an acute exacerbation of dyspnoea and/or cough and sputum.^2^ This poses a significant clinical issue because many guidelines require the presence of persistent airflow limitation after bronchodilation for confirming a COPD diagnosis.^1^ Indeed, our therapeutic decisions are based on clinical trials that included patients exhibiting persistent airflow limitation verified by a reduced postbronchodilator FEV_1_/FVC ratio, with a high burden of chronic respiratory symptoms.^28^

A key strength of this study is the comprehensive data on symptoms, exacerbations and spirometry, enabling the subclassification into both spirometric- (dnsCOPD, PRISm, sCOPD) and symptom and exacerbation history based GOLD A/B/E -groups to be used in conjunction.

Other strengths include the large cohort size, with almost 2500 dnsCOPD patients and more than 5000 PRISm patients in a real-life setting, as well as the estimated observation time of up to 163,000 patient years for mortality outcomes. The method of selecting dnsCOPD and PRISm patients within a cohort of patients with a physician diagnosis of COPD is a strength that differentiates this study from previous population-based cohorts. Another strength is the use of post-bronchodilation spirometric values. Previous cohort studies on PRISm trajectories and outcomes have frequently used spirometric values without bronchodilation, possibly leading to an overestimation of the number of PRISM patients within a cohort.^29^

Our study also has several limitations. Some previous cohort studies have labelled the group of physician diagnosed patients with normal spirometry as Pre-COPD.^23^ We have labelled it dnsCOPD instead, as the GOLD document definition of pre-COPD includes evidence of functional and/or structural changes. While some dnsCOPD patients might be pre-COPD, this category might also reflect other diseases. Second, our findings on dnsCOPD and PRISm reflects patients diagnosed with COPD by a physician in a clinical care setting and may not be generalisable to pre-COPD and PRISm identified in the general population.

Third, although the real-life cohort included over 45,000 COPD patients, a significant number of patients in SNAR were not included due to missing spirometry data. In order to address this potential selection-bias, an analysis of included and excluded patients was done, which showed only minor differences in BMI, smoking status and age. The impact of missing data on the validity of our findings is unlikely to be substantial. It is important to note that the missing data was primarily due to incomplete registration and not intrinsic patient characteristics, still it is possible that spirometry remains underutilized in the confirmation of COPD diagnosis within primary care in Sweden. Fourth, cause-of-death registers may have limited accuracy in some situations, in particular, COPD-exacerbations, which was part of our outcome-definition, might be under-reported as a cause of death.^30^ Fifth, some exacerbations might have been treated solely with antibiotics without the use of corticosteroids, or corticosteroids may have been prescribed for other indications than exacerbations, leading to misclassification and potential biased underestimation or overestimation of the incidence of exacerbations. This may in turn influence the A/B/E-classification of patients by classifying patients with two or more moderate exacerbations treated solely with antibiotics into the GOLD A group, and while the effect of this misclassification is likely small, it could somewhat weaken the observed association between the A/B/E-classification and the risk of the main outcomes. Nevertheless this method for defining moderate exacerbation has been validated in previous research.^11^ Sixth, we relied on real-life clinical data, with routine spirometry results obtained from primary and secondary care and were unable to assess the quality of the spirometries performed. Lastly, although the analyses were adjusted for different potential confounder variables to address confounding bias, our results might still be affected by residual confounding and results could be mediated by other factors. The results from our mediation analyses on comorbidities and medications suggested that the majority of the observed associations were independent of these mediators. Still, in clinical practice, these factors and other covariates are not considered to define group classification for treatment decisions.

The diagnosis of COPD has evolved over the last few decades, with the addition of symptoms, risk factors (particularly smoking), and exacerbations. Spirometry remains crucial for the initial diagnosis and assessment of severity. The recognition within the GOLD-document of pre-COPD and PRISm as clinical entities within physician diagnosed COPD is important to prompt research on treatment strategies for this important group of patients, which in our study represents 14% of the physician-diagnosed COPD population in current clinical practice in Sweden. Further research is crucial to assess the efficacy of current treatments, develop new approaches and bridge the knowledge gap regarding this clinically relevant patient population.

In conclusion, patients with dnsCOPD and PRISm are prevalent among patients with physician-diagnosis of COPD in primary and secondary care. These patients are symptomatic, might suffer from exacerbations and are commonly treated with inhaled therapy, at rates higher than GOLD stage 1 spirometrically confirmed COPD, despite spirometric findings that do not indicate obstructive lung disease. PRISm was associated with obesity, diabetes and cardiovascular disease. Compared to spirometrically confirmed COPD, both dnsCOPD and PRISm had generally lower risk of exacerbation, respiratory hospitalisation, respiratory and all-cause mortality, although all-cause hospitalisation and cardiovascular hospitalisation and cardiovascular mortality was similar in PRISm compared to spirometrically confirmed COPD. The GOLD A/B/E classification had high prognostic value, also in patients with dnsCOPD or PRISm.

## Contributors

All authors met the Lancet authorship criteria and participated significantly in the study. L.E.G.W.V. conceived and designed the analysis. O.W., C.S., A.L., F.N., L.E.G.W.V. collected the data and provided analysis tools. O.W. performed the analysis. O.W. and L.E.G.W.V. wrote the paper. All authors critically reviewed the manuscript. O. W. and L.E.G.W.V. had access to raw data, verified the data and shared the final responsibility for the decision to submit for publication.

## Data sharing statement

The health-data used in this register study is considered sensitive personal information and is thereby protected under common European Union and Swedish data-protection laws and cannot be shared for public use.

## Declaration of interests

O.W. reports a research grant from AstraZeneca paid to his institution and travel grants from the Swedish Heart-Lung Foundation and The Adlerbertska foundation. C.S. reports personal fees for lectures from GSK and AstraZeneca, institutional fees for manuscript writing from Chiesi and TEVA, support for attending meetings from AstraZeneca and personal fees for participation on advisory boards for AstraZeneca and GSK. H.B. has no conflicts of interest to declare. S.V. reports research grants from Sanofi, GSK and AstraZeneca paid to their institution and personal fees for lectures and educational events from GSK, AstraZeneca and Sanofi, a travel grant from PulmonX as well as personal fees for participation on advisory boards for GSK and Sanofi and unpaid participation in the Letten Prize board and the Scientify Research Communication award prize committee. A.L. reports personal fees for lectures from AstraZeneca and participation on advisory boards for GSK, AstraZeneca, Boehringer Ingelheim and Novartis. F.N. reports having received an unrestricted study grant from AstraZeneca and holding AstraZeneca shares. L.E.G.W.V. received the funding supporting the study from ALF grant and the Swedish Heart and Lung Foundation which directly supported this study and reports in addition; research grants from The Family Kamprad Foundation, Svensk Lungmedicinsk Förening, and AstraZeneca, and further discloses personal fees for lectures and educational events from GSK, AstraZeneca, Boehringer, Novartis, Chiesi, Resmed, Pulmonx, Grifols, and Sanofi.

## Appendix ASupplementary data

Supplementary Figs. E1–E7 and Tables E1–E9

STROBE-checklist_filled_PRISm_REV3
