# Supplementary material for: Risk of exacerbations, hospitalisation, and mortality in adults with physician-diagnosed chronic obstructive pulmonary disease with normal spirometry and adults with preserved ratio impaired spirometry in Sweden: retrospective analysis of data from a nationwide cohort study
Source: Lancet Reg Health Eur. 2025 May 14;54:101322. doi: 10.1016/j.lanepe.2025.101322 (PMC12143654; doi:10.1016/j.lanepe.2025.101322)

**Supplementary Material**

**Additional Information - The Swedish National Airway Register (SNAR)**

The Swedish National Airway Register (SNAR) is a national quality register that started in 2014 with the goal of improving the quality of care for asthma and COPD. The register collects data following a physician diagnosis of asthma or COPD, with patients records being either manually or automatically transferred from electronic medical records (EMRs). To date, over > 1000 clinics in Sweden are connected to SNAR, including, primary, secondary, tertiary, and inpatient care facilities with clinics from all of Sweden regions represented in the register. SNAR encompasses a wide range of variables including but not limited to smoking history, symptom scores, comorbidities, diagnostic investigations such as spirometry and prescribed treatments (both pharmacological and non-pharmacological). In Sweden, spirometry is primarily conducted following the ERS/ATS guidelines with both pre- and post-bronchodilator values being recorded in SNAR.

In terms of representativeness, SNAR includes data from a diverse range of healthcare settings and patients, ensuring high coverage of the COPD and asthma population in Sweden. However, not all primary care clinics are currently connected to the register, which may lead to a small underrepresentation of patients seeking care at primary health care facilities. This also means that people who do not seek healthcare or remain undiagnosed cannot be accounted for. Over the course of this study period, there have been incremental improvements in the way data is collected and defined within SNAR. These changes include the standardization of variable definitions, increased automation of data transfer from EMRs to the register, and the expansion of the number of participating clinics. Such measures have enhanced the quality, quantity and representativeness of the data over time.

For further information we would like to refer to “The Swedish National Airway Register – Development and Utility to date (doi 10.1080/20018525.2020.1833412) in which the register is further described and discussed extensively.

**Supplementary Material**

**Table E1:**

| **Comorbidity** | **ICD10 codes** |
| --- | --- |
| Myocardial infarction | I21-I24 |
| Stroke | I61, I63 |
| Heart failure | I50 |
| Atrial fibrillation | I48 |
| Coronary heart disease | I20-I25 |
| Hypertension | I10 or I109 |
| Peripheral artery disease | I702, I739 |
| Diabetes mellitus type 2 | E11 |

**Table E2: Drug therapy classes and ATC codes for respiratory inhaler therapy used in the Swedish Prescribed Drug Register.**

| **Drug therapy class** | **ATC codes for respiratory inhaler therapy** |
| --- | --- |
| SABA and/or SAMA | - R03AC02, Salbutamol - R03AC03, Terbutalin - R03BB01, Ipratropiumbromid - R03AL02, Salbutamol and ipratropiumbromid |
| LAMA | - R03BB01, Ipratropiumbromid - R03BB04, Tiotropiumbromid - R03BB05, Aclidiniumbromid - R03BB06, Glycopyrroniumbromid - R03BB07, Umeclidiniumbromid |
| LABA | - R03AC12, Salmeterol - R03AC13, Formoterol - R03AC18, Indacaterol - R03AC19, Olodaterol |
| ICS | - R03BA01, Beclometason - R03BA02, Budesonide - R03BA04, Betametason - R03BA05, Fluticason - R03BA07, Mometason - R03BA08, Ciklesonid |
| LABA/LAMA | - R03AL03, Vilanterol and Umeclidiniumbromid - R03AL04, Indacaterol and Glycopyrroniumbromid - R03AL05, Formoterol and Aclidiniumbromid - R03AL06, Olodaterol and Tiotropiumbromid - R03AL07, Formoterol and Glycopyrroniumbromid - R03AL10, Formoterol and Tiotropium |
| LABA/ICS | - R03AK06, Salmeterol and Fluticason - R03AK07, Formoterol and Budesonid - R03AK08, Formoterol and Beclometason - R03AK09, Formoterol and Mometason - R03AK10, Vilanterol and Fluticasonfuroat - R03AK11, Formoterol and Fluticason - R03AK12, Salmeterol and Budesonid - R03AK13, Salmeterol and Budesonid - R03AK14, Indakaterol and Mometason |
| LABA/LAMA/ICS | - R03AL08, Vilanterol, umeklidiniumbromid and flutikasonfuroat - R03AL09, Formoterol, Glycopyrroniumbromid and Beclometason - R03AL11, Formoterol, Glykopyrroniumbromid, Budesonide - R03AL12, Indakaterol, Glukopyrronium, Mometason |

**Table E3: Drug therapy classes and ATC codes for cardiovascular and diabetes therapy used in the Swedish Prescribed Drug Register.**

| **Drug therapy class** | **ATC codes** |
| --- | --- |
| Anticoagulation | B01 |
| Anti-arrhythmic | C01 |
| Diuretics | C03 |
| Betablocker | C07 |
| Calcium channel-blocker | C08 |
| ACE/ARB | C09 |
| Statin | C10 |
| Diabetes | A10 |
| Any cardiac drug | B01  C01  C02  C03  C07  C08  C09 |
| Any Metabolic drug | C10  A10 |
| Any Cardiometabolic drug | B01  C01  C02  C03  C07  C08  C09  A10 |

**Table E4: Crude and adjusted subdistribution hazard ratios for a first event of moderate exacerbation and all-cause, respiratory and cardiovascular hospitalisation and mortality among 45,653 patients aged >30 with complete spirometry and a physician diagnosis of COPD (dCOPD) registered in the Swedish National Airway Register, from 2014 to 2022, by dnsCOPD, PRISm and sCOPD.**

| **Moderate exacerbation*** | | | |  |  |  |  |
| --- | --- | --- | --- | --- | --- | --- | --- |
|  | **dnsCOPD** | **PRISm** | **sCOPD** |  |  |  |  |
| Crude SHR  (95%CI) | **0·68  (0·64-0·73)** | **0·86  (0·82-0·90)** | Ref |  |  |  |  |
| Adj SHR  (95%CI) | **0·69  (0·64-0·74)** | **0·85  (0·81-0·89)** | Ref |  |  |  |  |
| **Respiratory hospitalisation*** | | | | **Respiratory mortality*** | | | |
|  | **dnsCOPD** | **PRISm** | **sCOPD** |  | **dnsCOPD** | **PRISm** | **sCOPD** |
| Crude SHR  (95%CI) | **0·17  (0·10-0·28)** | **0·62  (0·57-0·67)** | Ref | Crude SHR  (95%CI) | **0·17  (0·10-0·28)** | **0·44  (0·35-0·55)** | Ref |
| Adj SHR  (95%CI) | **0·35  (0·30-0·41)** | **0·68  (0·62-0·73)** | Ref | Adj SHR  (95%CI) | **0·22  (0·13-0·37)** | **0·60  (0·48-0·75)** | Ref |
| **Cardiovascular hospitalisation*** | | | | **Cardiovascular mortality*** | | | |
|  | **dnsCOPD** | **PRISm** | **sCOPD** |  | **dnsCOPD** | **PRISm** | **sCOPD** |
| Crude SHR  (95%CI) | **0·78  (0·64-0·96)** | 0·99  (0·87-1·13) | Ref | Crude SHR  (95%CI) | **0·37  (0·17-0·77)** | **0·65  (0·44-0·97)** | Ref |
| Adj SHR  (95%CI) | 0·85  (0·69-1·04) | 1·06 (0·93-1·20) | Ref | Adj SHR  (95%CI) | **0·41  (0·19-0·86)** | 0·73 (0·49-1·08) | Ref |
| **All-cause hospitalisation*** | | | | **All-cause mortality** | | | |
|  | **dnsCOPD** | **PRISm** | **sCOPD** |  | **dnsCOPD** | **PRISm** | **sCOPD** |
| Crude SHR  (95%CI) | **0·81  (0·76-0·86)** | 0·97  (0·93-1·01) | Ref | Crude HR  (95%CI) | **0·51  (0·44-0·58)** | **0·79  (0·73-0·85)** | Ref |
| Adj SHR  (95%CI) | **0·87  (0·83-0·94)** | 0·99  (0·95-1·04) | Ref | Adj HR  (95%CI) | **0·59  (0·52-0·68)** | 0·94  (0·87-1·02) | Ref |

Notes: SHR: Subdistribution Hazard Ratio; CI: confidence interval; HR: Hazard Ratio, dnsCOPD = physician diagnosed COPD with normal spirometry,
sCOPD = Spirometrically confirmed COPD,
Adj· = Adjustment for age, sex, BMI, smoking status, previous cardiovascular disease and previous cardioprotective medications; * Death from another cause was considered as a competing risk.

**Table E5: Crude and adjusted subdistribution hazard ratios for moderate exacerbation and all-cause, respiratory and cardiovascular hospitalisation and mortality for dnsCOPD with sCOPD stage 1 as reference.**

| **Moderate exacerbations*** | | |  |  |  |
| --- | --- | --- | --- | --- | --- |
|  | **dnsCOPD** | **sCOPD stage 1** |  |  |  |
| Crude SHR  (95%CI) | **1·11  (1·02 -1·21)** | Ref |  |  |  |
| Adj SHR  (95%CI) | 1·07  (0·98-1·17) | Ref |  |  |  |
| **Respiratory hospitalisation*** | | | **Respiratory mortality*** | | |
|  | **dnsCOPD** | **sCOPD stage 1** |  | **dnsCOPD** | **sCOPD stage 1** |
| Crude SHR  (95%CI) | 1·15  (0·94-1·40) | Ref | Crude SHR  (95%CI) | 0·92  (0·47-1·77) | Ref |
| Adj SHR  (95%CI) | 1·20  (0·98- 1·46) | Ref | Adj SHR  (95%CI) | 1·25  (0·66-2·36) | Ref |
| **Cardiovascular hospitalisation*** | | | **Cardiovascular mortality*** | | |
|  | **dnsCOPD** | **sCOPD stage 1** |  | **dnsCOPD** | **sCOPD stage 1** |
| Crude SHR  (95%CI) | 0·97  (0·75-1·24) | Ref | Crude SHR  (95%CI) | 0·44  (0·19-1·02) | Ref |
| Adj SHR  (95%CI) | 1·05  (0·81-1·36) | Ref | Adj SHR  (95%CI) | 0·51  (0·22-1·24) | Ref |
| **All-cause hospitalisation*** | | | **All-cause mortality** | | |
|  | **dnsCOPD** | **sCOPD stage 1** |  | **dnsCOPD** | **sCOPD stage 1** |
| Crude SHR  (95%CI) | **1·11  (1·03-1·20)** | Ref | Crude HR  (95%CI) | 1·01  (0·85-1·20) | Ref |
| Adj SHR  (95%CI) | **1·13 (1·05-1·22)** | Ref | Adj HR  (95%CI) | **1·21  (1·01-1·44)** | Ref |

Notes: SHR: Subdistribution Hazard Ratio, CI: confidence interval, HR: Hazard Ratio,
sCOPD = spirometrically confirmed COPD,
dnsCOPD = physician diagnosed COPD with normal spirometry,
 Adj· = Adjustment for age, sex, BMI, smoking status, cardiovascular disease and cardiovascular medications

* Death from another cause was considered as a competing risk.

**Table E6: Crude and adjusted subdistribution hazard ratios for moderate exacerbation and all-cause, respiratory and cardiovascular hospitalisation and mortality for PRISm with sCOPD stage 2+ as reference.**

| **Moderate exacerbations*** | | |  |  |  |
| --- | --- | --- | --- | --- | --- |
|  | **PRISm** | **sCOPD stage 2+** |  |  |  |
| Crude SHR  (95%CI) | **0·82  (0·79-0·86)** | Ref |  |  |  |
| Adj SHR  (95%CI) | **0·82  (0·78-0·85)** | Ref |  |  |  |
| **Respiratory hospitalisation*** | | | **Respiratory mortality*** | | |
|  | **PRISm** | **sCOPD stage 2+** |  | **PRISm** | **sCOPD stage 2+** |
| Crude SHR  (95%CI) | **0·57  (0·52-0·62)** | Ref | Crude SHR  (95%CI) | **0·40  (0·32-0·50)** | Ref |
| Adj SHR  (95%CI) | **0·63  (0·58-0·69)** | Ref | Adj SHR  (95%CI) | **0·55  (0·44-0·70)** | Ref |
| **Cardiovascular hospitalisation*** | | | **Cardiovascular mortality*** | | |
|  | **PRISm** | **sCOPD stage 2+** |  | **PRISm** | **sCOPD stage 2+** |
| Crude SHR  (95%CI) | 0·97  (0·85-1·10) | Ref | Crude SHR  (95%CI) | **0·64  (0·43-0·95)** | Ref |
| Adj SHR  (95%CI) | 1·04 (0·91-1·18) | Ref | Adj SHR  (95%CI) | 0·71  (0·48-1·05) | Ref |
| **All-cause hospitalisation*** | | | **All-cause mortality** | | |
|  | **PRISm** | **sCOPD stage 2+** |  | **PRISm** | **sCOPD stage 2+** |
| Crude SHR  (95%CI) | **0·94  (0·90-0·98)** | Ref | Crude HR  (95%CI) | **0·75  (0·69-0·81)** | Ref |
| Adj SHR  (95%CI) | 0·97  (0·93-1·01) | Ref | Adj HR  (95%CI) | **0·90  (0·83-0·97)** | Ref |

Notes: SHR: Subdistribution Hazard Ratio, CI: confidence interval, HR: Hazard Ratio,
sCOPD = spirometrically confirmed COPD
Adj· = Adjustment for age, sex, BMI, smoking status, cardiovascular disease and cardiovascular medications

* Death from another cause was considered as a competing risk.

**Table E7: Crude and adjusted subdistribution hazard ratios for moderate exacerbation and all-cause, respiratory and cardiovascular hospitalisation and mortality for dnsCOPD, PRISm and sCOPD further stratified according to A, B, E. sCOPD GOLD E as Reference.**

|  | **Moderate exacerbation** | |  | | | |
| --- | --- | --- | --- | --- | --- | --- |
|  | Crude SHR  (95%CI) | Adj SHR  (95%CI) |  |  |  |  |
| **dnsCOPD GOLD A** | 0·15 (0·14-0·18) | 0·16 (0·14-0·18) |  |  |  |  |
| **PRISm GOLD A** | 0·18 (0·16-0·20) | 0·18 (0·17-0·20) |  |  |  |  |
| **sCOPD A** | 0·20 (0·19-0·21) | 0·20 (0·19-0·21) |  |  |  |  |
| **dnsCOPD GOLD B** | 0·20 (0·19-0·23) | 0·21 (0·19-0·23) |  |  |  |  |
| **PRISm GOLD B** | 0·25 (0·23-0·26) | 0·25 (0·23-0·27) |  |  |  |  |
| **sCOPD GOLD B** | 0·29 (0·28-0·30) | 0·30 (0·29-0·31) |  | | | |
| **dnsCOPD GOLD E** | 0·76 (0·63-0·91) | 0·75 (0·63-0·90) |  | | | |
| **PRISm GOLD E** | 0·93 (0·84-1·03) | 0·92 (0·83-1·02) |  | | | |
| **sCOPD GOLD E** | Ref | Ref |  | | | |
|  | **All-cause hospitalisation** | | **Respiratory hospitalisation*** | | **Cardiovascular hospitalisation*** | |
|  | Crude SHR  (95%CI) | Adj SHR  (95%CI) | Crude SHR  (95%CI) | Adj SHR  (95%CI) | Crude SHR (95%CI) | Adj SHR  (95%CI) |
| **dnsCOPD GOLD A** | 0·39 (0·35-0·43) | 0·48 (0·43-0·54) | 0·09 (0·06-0·12) | 0·12 (0·09-0·16) | 0·36 (0·24-0·55) | 0·46 (0·30-0·69) |
| **PRISm GOLD A** | 0·40 (0·37-0·44) | 0·48 (0·44-0·53) | 0·11 (0·09-0·14) | 0·15 (0·12-0·18) | 0·60 (0·46-0·78) | 0·74 (0·57-0·97) |
| **sCOPD GOLD A** | 0·40 (0·38-0·41) | 0·46 (0·44-0·48) | 0·16 (0·15-0·17) | 0·19 (0·18-0·21) | 0·60 (0·52-0·68) | 0·70 (0·62-0·80) |
| **dnsCOPD GOLD B** | 0·46 (0·42-0·50) | 0·54 (0·50-0·59) | 0·13 (0·10-0·16) | 0·16 (0·13-0·20) | 0·62 (0·48-0·82) | 0·74 (0·56-0·97) |
| **PRISm GOLD B** | 0·56 (0·53-0·59) | 0·64 (0·61-0·68) | 0·22 (0·20-0·25) | 0·28 (0·25-0·31) | 0·70 (0·58-0·83) | 0·82 (0·68-0·99) |
| **sCOPD GOLD B** | 0.57 (0.55-0.59) | 0.63 (0.61-0.65) | 0·36 (0·34-0·38) | 0·41 (0·39-0·43) | 0·66 (0·59-0·74) | 0·73 (0·65-0·81) |
| **dnsCOPD GOLD E** | 0.75 (0.64-0.88) | 0.78 (0.66-0.91) | 0·36 (0·27-0·49) | 0·40 (0·30-0·54) | 0·76 (0·45-1·30) | 0·80 (0·47-1·36) |
| **PRISm GOLD E** | 0.86 (0.78-0.95) | 0.90 (0.81-0.99) | 0·62 (0·53-0·72) | 0·70 (0·60-0·81) | 0·84 (0·61-1·15) | 0·90 (0·66-1·24) |
| **sCOPD GOLD E** | Ref | Ref | Ref | Ref | Ref | Ref |
|  | **All-cause mortality** | | **Respiratory mortality*** | | **Cardiovascular mortality*** | |
|  | Crude HR  (95%CI) | Adj HR  (95%CI) | Crude SHR  (95%CI) | Adj SHR  (95%CI) | Crude SHR (95%CI) | Adj SHR  (95%CI) |
| **dnsCOPD A** | 0·22 (0·17-0·29) | 0·33 (0·26-0·42) | 0·02 (0·01-0·10) | 0·04 (0·01-0·15) | 0·26 (0·08-0·82) | 0·37 (0·12-1·18) |
| **PRISm A** | 0·27 (0·23-0·33) | 0·42 (0·35-0·50) | 0·08 (0·04-0·14) | 0·14 (0·08-0·25) | 0·31 (0·13-0·71) | 0·43 (0·19-0·99) |
| **sCOPD A** | 0·29 (0·27-0·31) | 0·38 (0·35-0·41) | 0·08 (0·06-0·10) | 0·10 (0·08-0·13) | 0·34 (0·24-0·48) | 0·45 (0·31-0·63) |
| **dnsCOPD B** | 0·26 (0·22-0·32) | 0·36 (0·30-0·44) | 0·07 (0·04-0·14) | 0·11 (0·06-0·22) | 0·17 (0·05-0·53) | 0·22 (0·07-0·69) |
| **PRISm B** | 0·41 (0·37-0·45) | 0·59 (0·53-0·66) | 0·13 (0·10-0·18) | 0·23 (0·16-0·31) | 0·40 (0·24-0·68) | 0·54 (0·32-0·91) |
| **sCOPD B** | 0·52 (0·49-0·55) | 0·62 (0·59-0·66) | 0·31 (0·28-0·35) | 0·38 (0·34-0·43) | 0·59 (0·45-0·76) | 0·69 (0·53-0·90) |
| **dnsCOPD E** | 0·46 (0·33-0·64) | 0·53 (0·38-0·73) | 0·12 (0·04-0·38) | 0·16 (0·05-0·52) | 0·29 (0·04-2·10) | 0·31 (0·04-2·27) |
| **PRISm E** | 0·76 (0·65-0·89) | 0·92 (0·78-1·07) | 0·41 (0·28-0·60) | 0·57 (0·39-0·84) | 0·42 (0·15-1·14) | 0·48 (0·18-1·29) |
| **sCOPD E** | Ref | Ref | Ref | Ref | Ref | Ref |

SHR: subdistribution hazard ratio, HR: hazard ratio CI: confidence interval, Adj. = Adjustment for age, sex, smoking status, previous cardiovascular disease and previous cardioprotective medications * Death from another cause considered as a competing risk

**Table E8: Mediation analysis on 4 main outcomes with baseline comorbidity as mediator.**

| **Outcome** | **Effect type** | **Estimate (β)** | **95%CI** | **p-value** |
| --- | --- | --- | --- | --- |
| Exacerbations | ACME (average) | 0.002 | 0.00128 - 0.00 | <2e-16 |
|  | ADE (average) | -0.074 | -0.09 - 0.06 | <2e-16 |
|  | Prop. Mediated | -0.0279 | -0.04 – (-0.02) | <2e-16 |
| Respiratory Mortality | ACME (average) | .00071 | 0.00044 - 0.00 | <2e-16 |
|  | ADE (average) | -0.024 | -0.027- - 0.02 | <2e-16 |
|  | Prop. Mediated | -0.031 | -0.045 - -0.02 | <2e-16 |
| CVD Mortality | ACME (average) | 0.0002 | 0.00012 - 0.00 | <2e-16 |
|  | ADE (average) | -0.0038 | -0.0055- 0.00 | <2e-16 |
|  | Prop. Mediated | -0.055 | -0.12 - -0.03 | <2e-16 |
| All-Cause Mortality | ACME (average) | 0.0039 | 0.0027 - 0.01 | <2e-16 |
|  | ADE (average) | -0.061 | -0.069 - -0.05 | <2e-16 |
|  | Prop. Mediated | -8% | -10% - -5% | <2e-16 |

*(ACME = Average Causal Mediation Effect, ADE = Average Direct Effect, Prop. Mediated = Proportion of effect explained by the mediator)*

**Table E9: Mediation analysis on 4 main outcomes with baseline cardioprotective medications as mediator.**

| **Outcome** | **Effect type** | **Estimate (β)** | **95%CI** | **p-value** |
| --- | --- | --- | --- | --- |
| Exacerbations | ACME (average) | 0.0011 | 0.0007 - 0.00 | <2e-16 |
|  | ADE (average) | -0.073472 | -0.083 - -0.06 | <2e-16 |
|  | Prop. Mediated | -1.6% | -2.7% - -1% | <2e-16 |
| Respiratory Mortality | ACME (average) | 0.00037 | 0.00024 - 0.00 | <2e-16 |
|  | ADE (average) | -0.023 | -0.026 - -0.02 | <2e-16 |
|  | Prop. Mediated | -1.6% | -2.5% - -1% | <2e-16 |
| CVD Mortality | ACME (average) | 0.00017 | 0.0001- 0.00 | <2e-16 |
|  | ADE (average) | -0.0038 | -0.0056 - 0.00 | <2e-16 |
|  | Prop. Mediated | -4.7% | -12% - -2% | <2e-16 |
| All-Cause Mortality | ACME (average) | 0.0026 | 0.0017 - 0.00 | <2e-16 |
|  | ADE (average) | -0.059 | -0.067 - -0.05 | <2e-16 |
|  | Prop. Mediated | -4.5% | -7.5% - -3% | <2e-16 |

**Figure E1: Cohort inclusion, covariate assessment and follow-up**

**
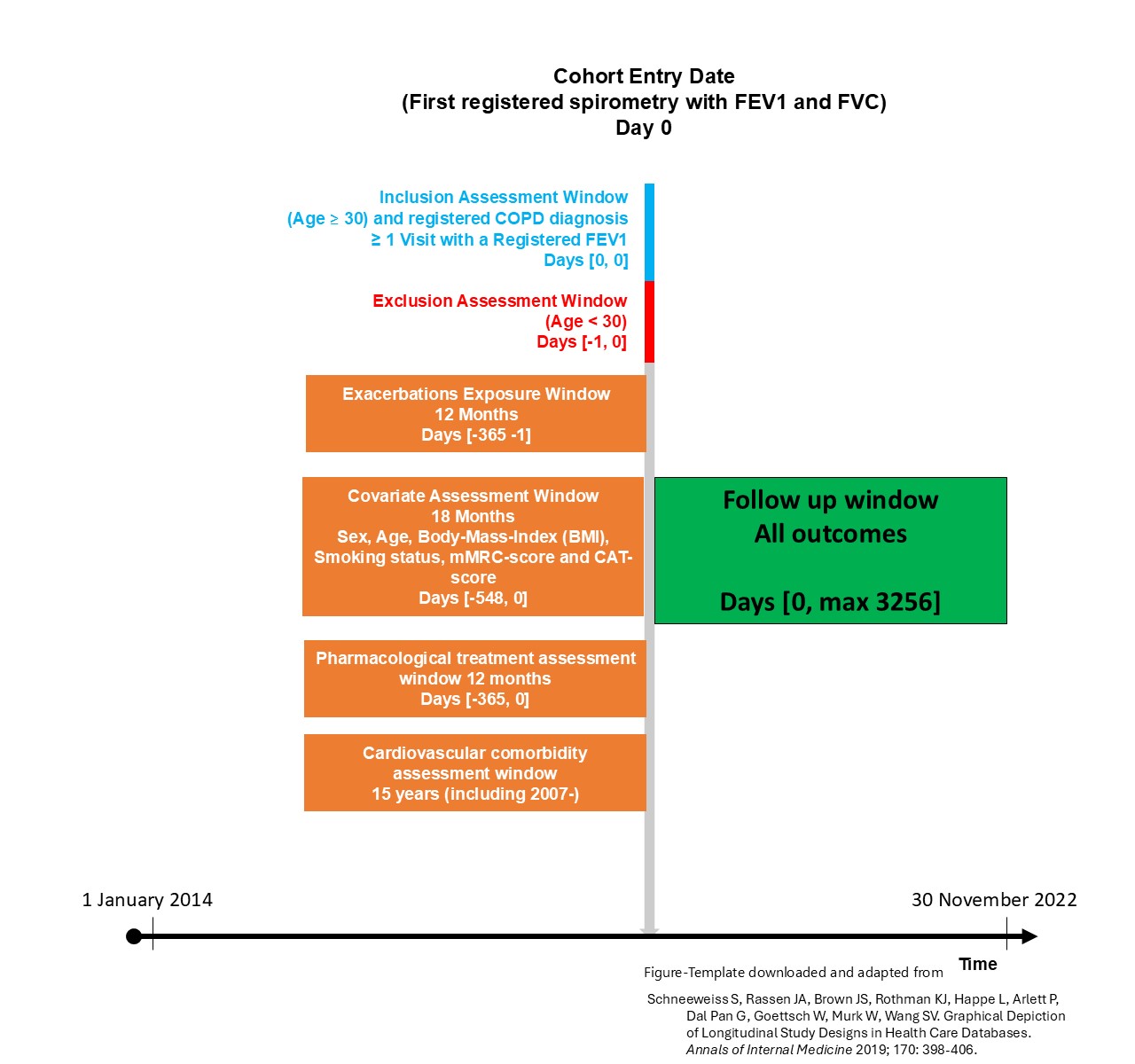
**

**Respiratory Hospitalisations**

**Figure E2 Cumulative probability of first respiratory hospitalisation in physician diagnosed COPD patients with dnsCOPD, PRISm and COPD (Panel A), by dnsCOPD, PRISm, COPD stage 1 and COPD stages 2,3 and 4 (Panel B) and by dnsCOPD, PRISm and COPD further stratified for subgroups A, B, and E (Panel C).**

**A B**


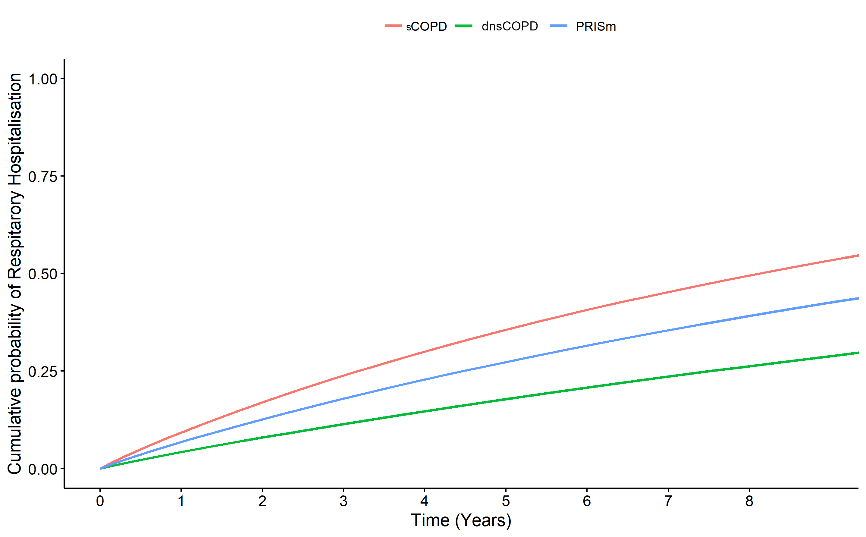

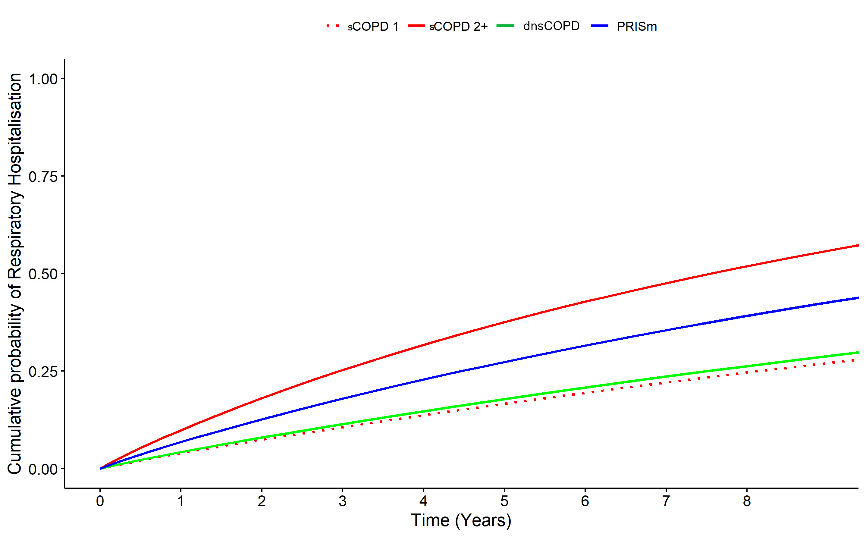


**C**

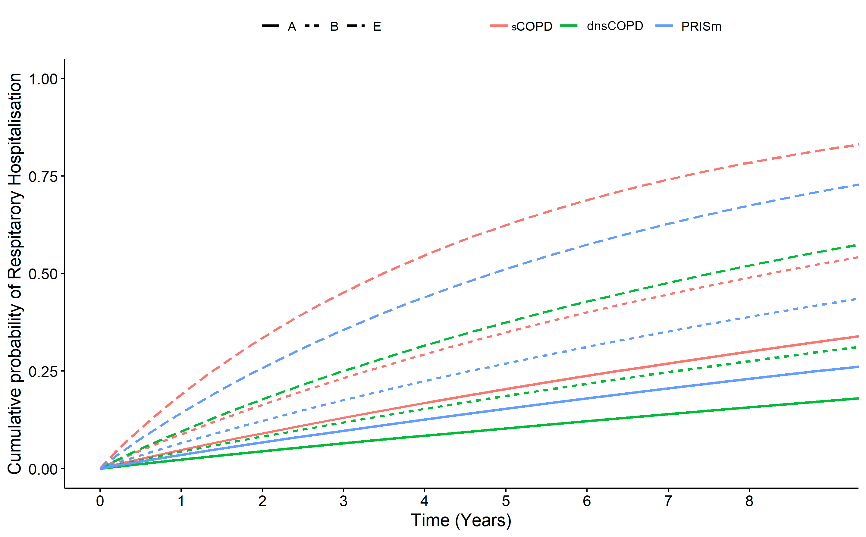


**Respiratory Mortality**

**Figure E3 Cumulative probability of respiratory mortality in physician diagnosed COPD patients with dnsCOPD, PRISm and COPD (Panel A), by dnsCOPD, PRISm, COPD stage 1 and COPD stages 2,3 and 4 (Panel B) and by dnsCOPD, PRISm and COPD further stratified for subgroups A, B, and E (Panel C).**

**A B**


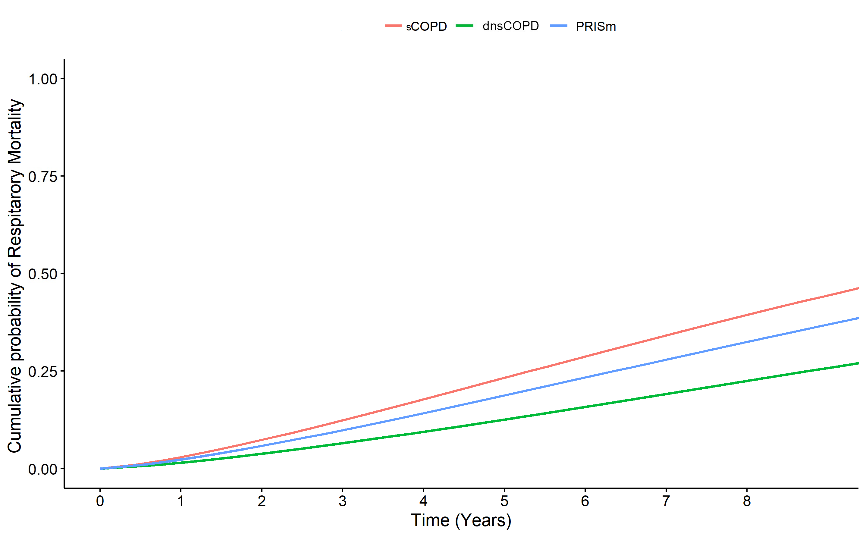

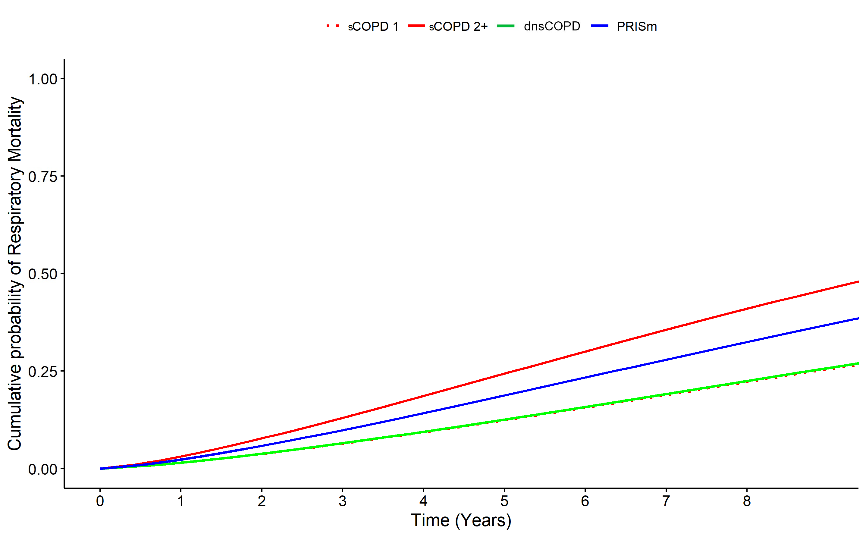


**C**

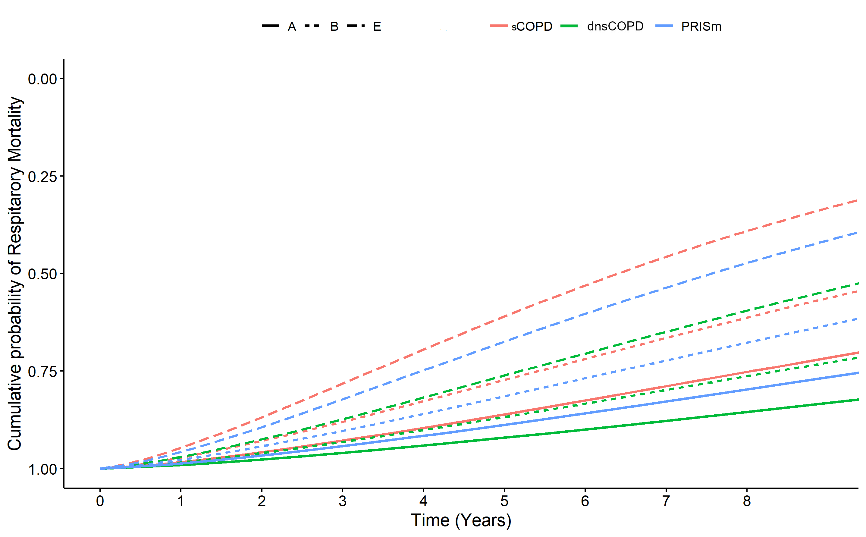


**Cardiovascular hospitalisations**

**Figure E4 Cumulative probability of first cardiovascular hospitalisation in physician diagnosed COPD patients with dnsCOPD, PRISm and COPD (Panel A), by dnsCOPD, PRISm, COPD stage 1 and COPD stages 2,3 and 4 (Panel B) and by dnsCOPD, PRISm and COPD further stratified for subgroups A, B, and E (Panel C).**

**A B**


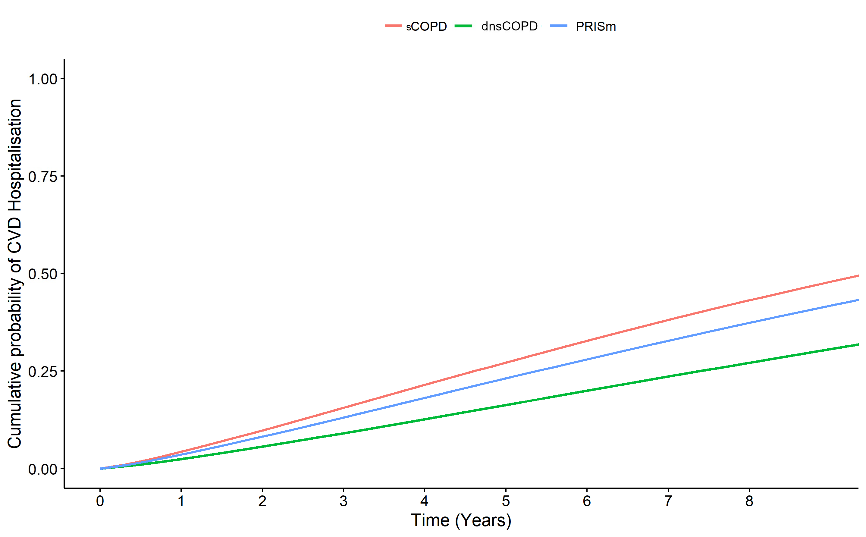

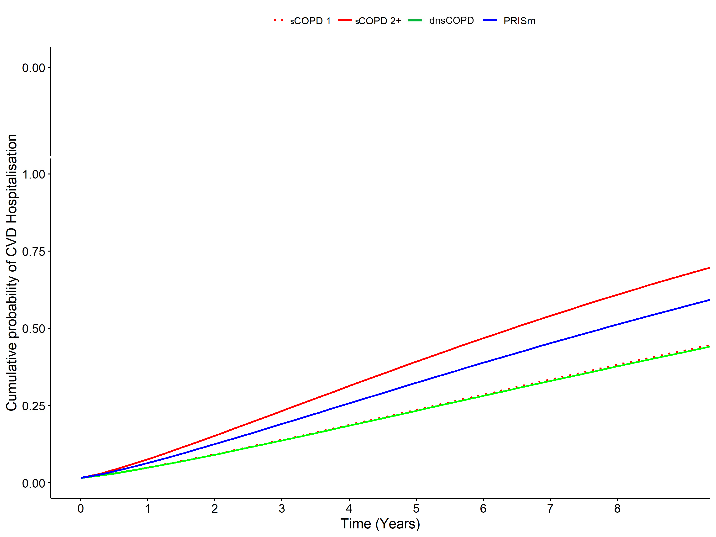


**C**

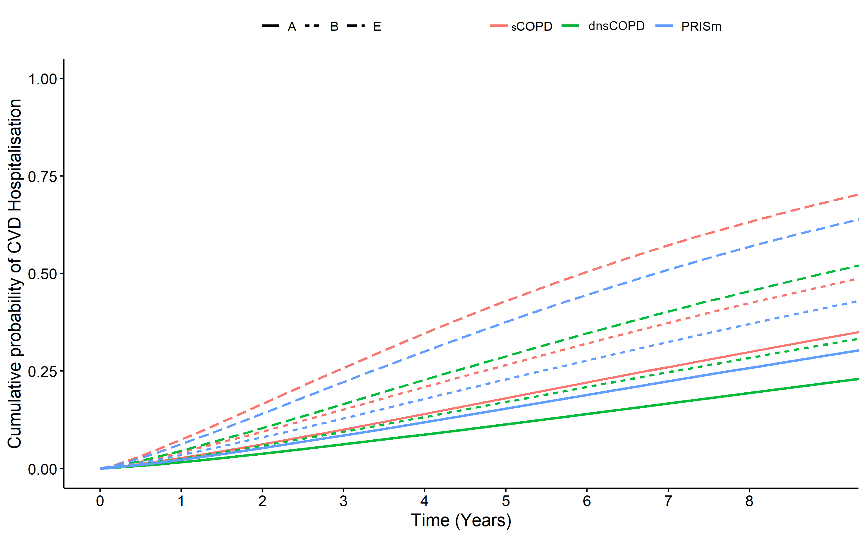


**Cardiovascular mortality**

**Figure E5 Cumulative probability of cardiovascular mortality in physician diagnosed COPD patients with dnsCOPD, PRISm and COPD (Panel A), by dnsCOPD, PRISm, COPD stage 1 and COPD stages 2,3 and 4 (Panel B) and by dnsCOPD, PRISm and COPD further stratified for subgroups A, B, and E (Panel C).**

**A B**


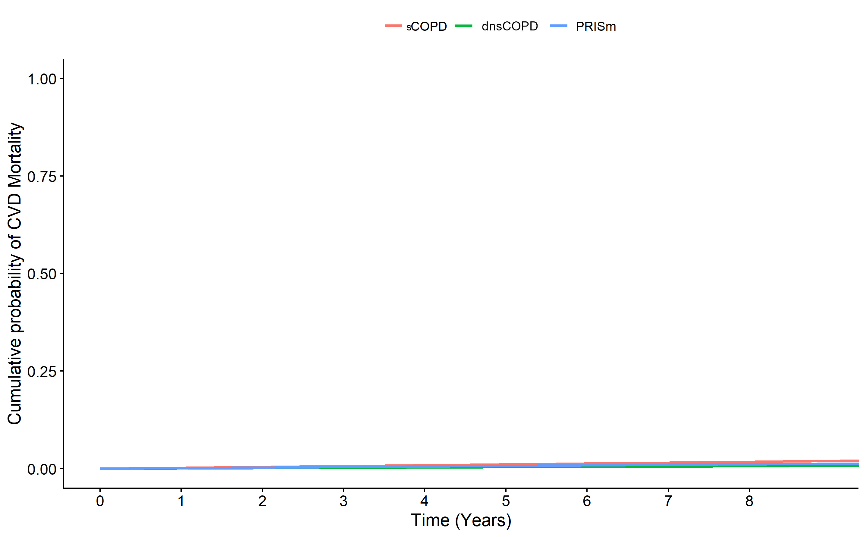

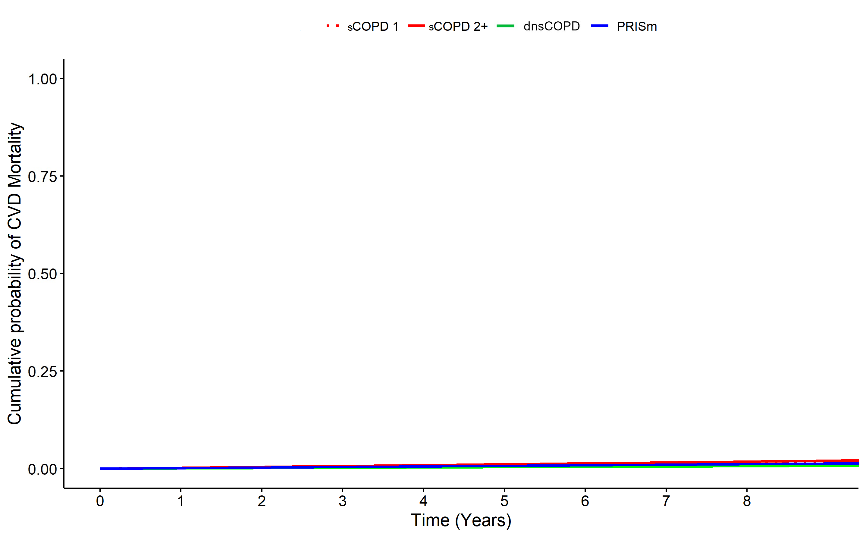


**C**

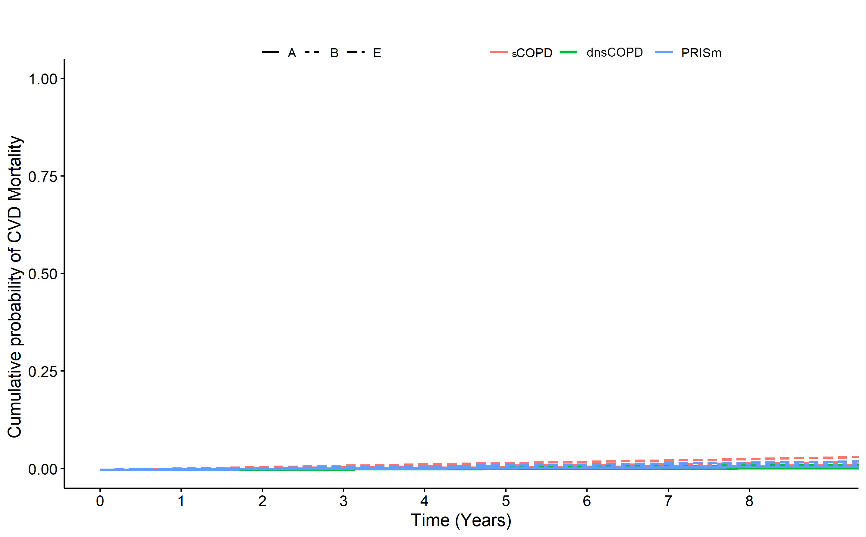


**All Cause Hospitalisation**

**Figure E6 Cumulative probability of first hospitalisation for any cause in physician diagnosed COPD patients with dnsCOPD, PRISm and COPD (Panel A), by dnsCOPD, PRISm, COPD stage 1 and COPD stages 2,3 and 4 (Panel B) and by dnsCOPD, PRISm and COPD further stratified for subgroups A, B, and E (Panel C).**

**A B**


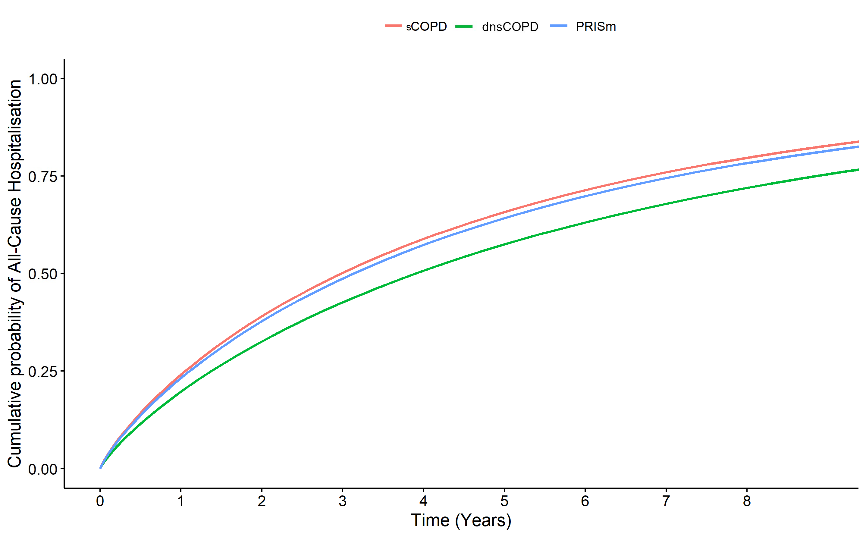

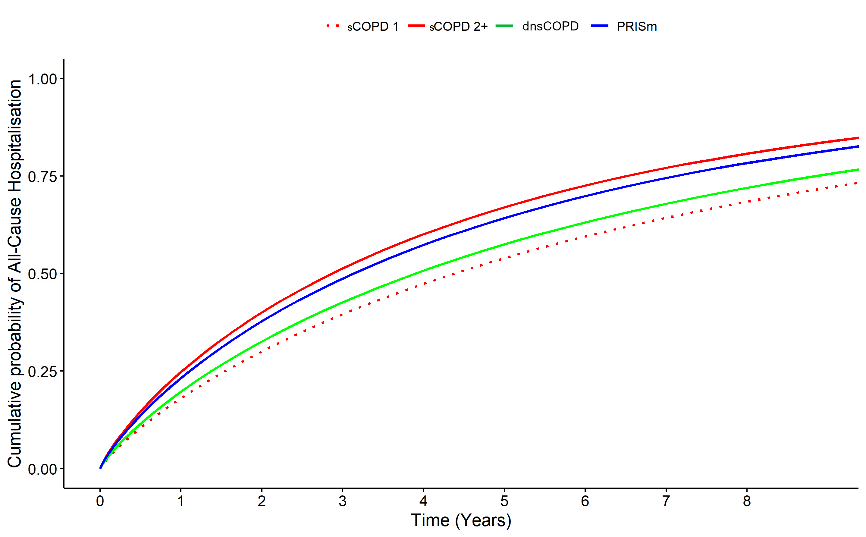


**C**

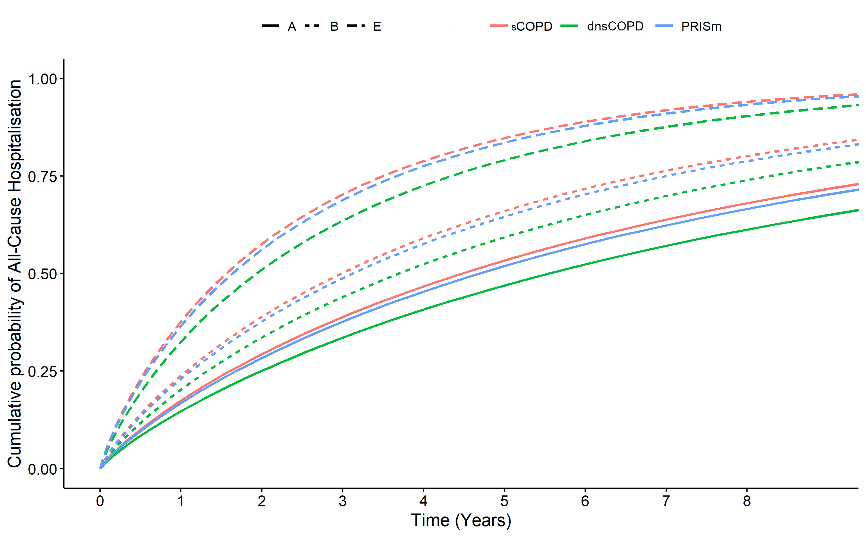


**All Cause Mortality**

**Figure E7 Cumulative probability of all-cause mortality in physician diagnosed COPD patients with dnsCOPD, PRISm and COPD (Panel A), by dnsCOPD, PRISm, COPD stage 1 and COPD stages 2,3 and 4 (Panel B) and by dnsCOPD, PRISm and COPD further stratified for subgroups A, B, and E (Panel C).**

**A B**


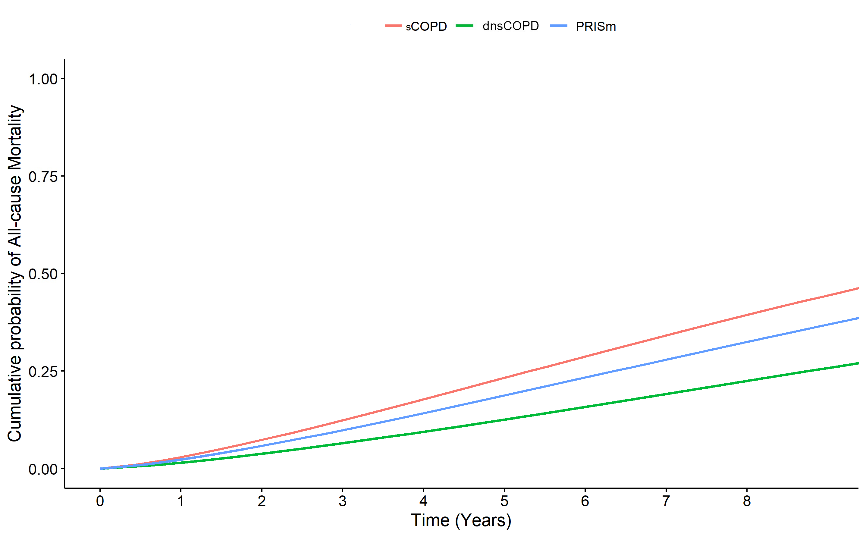

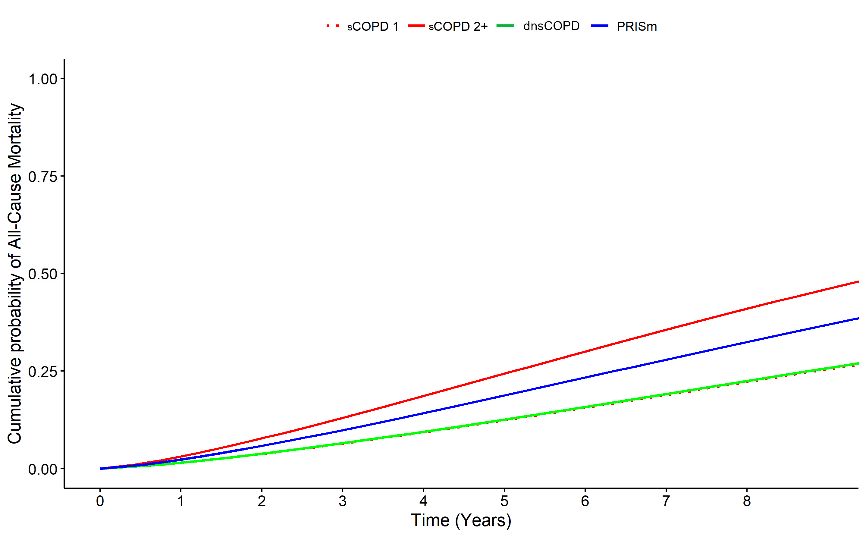


**C**

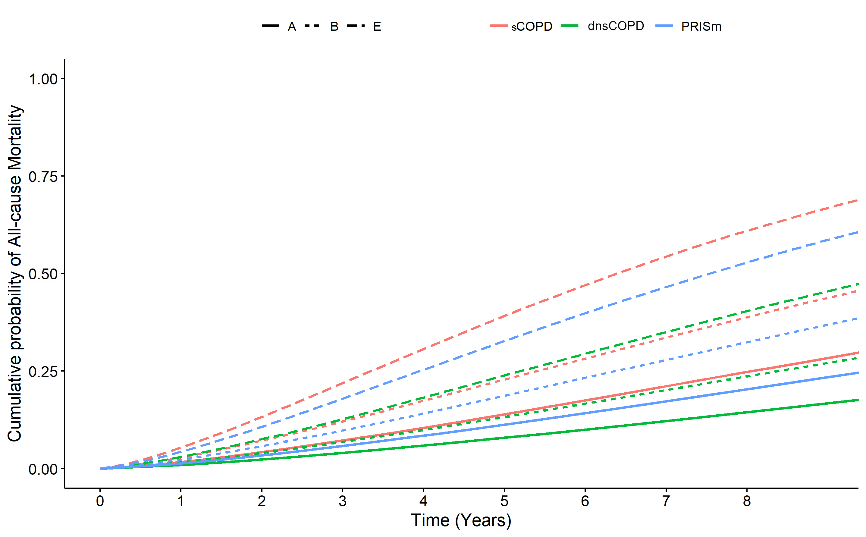

Supplement: Supplementary Figs. E1–E7 and Tables E1–E9 [file mmc1.docx]
