# Supplementary material for: Risk of exacerbations, hospitalisation, and mortality in adults with physician-diagnosed chronic obstructive pulmonary disease with normal spirometry and adults with preserved ratio impaired spirometry in Sweden: retrospective analysis of data from a nationwide cohort study
Source: Lancet Reg Health Eur. 2025 May 14;54:101322. doi: 10.1016/j.lanepe.2025.101322 (PMC12143654; doi:10.1016/j.lanepe.2025.101322)
Supplement: STROBE-checklist_filled_PRISm_REV3 [file mmc2.pdf]

STROBE Statement—checklist of items that should be included in reports of observational studies

	Item No.	Recommendation	Page No.	Relevant text from manuscript
**Title and abstract**	1	(*a*) Indicate the study's design with a commonly used term in the title or the abstract	1	Risk of exacerbation, hospitalisation, and mortality in physician diagnosed COPD with normal spirometry and PRISm patients: Insights from a routine clinical practice adult COPD **cohort** in the Swedish National Airway **Register**
		(*b*) Provide in the abstract an informative and balanced summary of what was done and what was found	2-3	
**Introduction**				
Background/rationale	2	Explain the scientific background and rationale for the investigation being reported	4	
Objectives	3	State specific objectives, including any prespecified hypotheses	4	We aimed to: first, identify and characterize patients with spirometrically pre-COPD and PRISm within the cohort of physician-diagnosed COPD in SNAR; second, to stratify them further according to symptoms and exacerbations risk using the GOLD ABE classification; and third, to assess differences between pre-COPD, PRISm, and spirometrically confirmed COPD patients in relation to future risk for exacerbations, and cause-specific hospitalisations and mortality.
**Methods**				
Study design	4	Present key elements of study design early in the paper	5	National cohort study, the study period, the inclusion criteria are introduced in the first paragraph

Setting	5	Describe the setting, locations, and relevant dates, including periods of recruitment, exposure, follow-up, and data collection	5-7	
Participants	6	(*a*) *Cohort study*—Give the eligibility criteria, and the sources and methods of selection of participants. Describe methods of follow-up	4	
Variables	7	Clearly define all outcomes, exposures, predictors, potential confounders, and effect modifiers. Give diagnostic criteria, if applicable	5-7	Exposure groups and their criteria, covariates and outcomes are described
Data sources/ measurement	8*	For each variable of interest, give sources of data and details of methods of assessment (measurement). Describe comparability of assessment methods if there is more than one group	5-6	The Swedish National Airway Register SNAR, the National Patient Register (NPR), the National Cause of Death Register (NCDR), and the National Prescribed Drug Register (NPDR) to obtain relevant data
Bias	9	Describe any efforts to address potential sources of bias	8, 15	
Study size	10	Explain how the study size was arrived at	5	All COPD patients recorded in SNAR who met the predefined inclusion criteria were included, and the size of the cohort reflects the available data on patients in SNAR within the study period.

Continued on next page

Quantitative variables	11	Explain how quantitative variables were handled in the analyses. If applicable, describe which groupings were chosen and why	7	
Statistical methods	12	(*a*) Describe all statistical methods, including those used to control for confounding	7-8	
		(*b*) Describe any methods used to examine subgroups and interactions	7-8	
		(*c*) Explain how missing data were addressed	8	
		(*d*) *Cohort study*—If applicable, explain how loss to follow-up was addressed		
		(*e*) Describe any sensitivity analyses	8	Mediation analysis on cardiovascular comorbidities and medications
**Results**				
Participants	13*	(a) Report numbers of individuals at each stage of study—eg numbers potentially eligible, examined for eligibility, confirmed eligible, included in the study, completing follow-up, and analysed	*Supplementary Figure E1*	
		(b) Give reasons for non-participation at each stage	*Supplementary Figure E1*	
		(c) Consider use of a flow diagram	*Supplementary Figure E1*	
Descriptive data	14*	(a) Give characteristics of study participants (eg demographic, clinical, social) and information on exposures and potential confounders	*Table 1*	
		(b) Indicate number of participants with missing data for each variable of interest	*Table 1*	
		(c) *Cohort study*—Summarise follow-up time (eg, average and total amount)	*Table 3*	
Outcome data	15*	*Cohort study*—Report numbers of outcome events or summary measures over time	*Table 3*	
Main results	16	(*a*) Give unadjusted estimates and, if applicable, confounder-adjusted estimates and their precision (eg, 95% confidence interval). Make clear which confounders were adjusted for and why they were included	*Figure 5-6*	
		(*b*) Report category boundaries when continuous variables were categorized		
		(*c*) If relevant, consider translating estimates of relative risk into absolute risk for a meaningful time period		

Continued on next page

Other analyses	17	Report other analyses done—eg analyses of subgroups and interactions, and sensitivity analyses	12	Results of mediation analysis
**Discussion**				
Key results	18	Summarise key results with reference to study objectives	13	Discussion Paragraph 1
Limitations	19	Discuss limitations of the study, taking into account sources of potential bias or imprecision. Discuss both direction and magnitude of any potential bias	15-16	Paragraphs 9-11 of discussion
Interpretation	20	Give a cautious overall interpretation of results considering objectives, limitations, multiplicity of analyses, results from similar studies, and other relevant evidence	16	
Generalisability	21	Discuss the generalisability (external validity) of the study results	15	
**Other information**				
Funding	22	Give the source of funding and the role of the funders for the present study and, if applicable, for the original study on which the present article is based	9	

*Give information separately for cases and controls in case-control studies and, if applicable, for exposed and unexposed groups in cohort and cross-sectional studies.

Note: An Explanation and Elaboration article discusses each checklist item and gives methodological background and published examples of transparent reporting. The STROBE checklist is best used in conjunction with this article (freely available on the Web sites of PLoS Medicine at http://www.plosmedicine.org/, Annals of Internal Medicine at http://www.annals.org/, and Epidemiology at http://www.epidem.com/). Information on the STROBE Initiative is available at www.strobe-statement.org.
